# Experience and Reporting of Postnatal Depression Across Cultures: A Comparison Using Anchoring Vignettes of Mothers in the United Kingdom and India

**DOI:** 10.1093/aje/kwad182

**Published:** 2023-09-01

**Authors:** Matthew Bluett-Duncan, Andrew Pickles, Prabha S Chandra, Jonathan Hill, M Thomas Kishore, Veena Satyanarayana, Helen Sharp

**Keywords:** anchoring vignettes, cross-cultural comparison, data harmonization, differential item functioning, Edinburgh Postnatal Depression Scale, global mental health, postnatal depression, response style

## Abstract

Postnatal mental health is often assessed using self-assessment questionnaires in epidemiologic research. Differences in response style, influenced by language, culture, and experience, may mean that the same response may not have the same meaning in different settings. These differences need to be identified and accounted for in cross-cultural comparisons. Here we describe the development and application of anchoring vignettes to investigate the cross-cultural functioning of the Edinburgh Postnatal Depression Scale (EPDS) in urban community samples in India (*n* = 549) and the United Kingdom (*n* = 828), alongside a UK calibration sample (*n* = 226). Participants completed the EPDS and anchoring vignettes when their children were 12–24 months old. In an unadjusted item-response theory model, UK mothers reported higher depressive symptoms than Indian mothers (*d* = 0.48, 95% confidence interval: 0.358, 0.599). Following adjustment for differences in response style, these positions were reversed (*d* = −0.25, 95% confidence interval: −0.391, −0.103). Response styles vary between India and the United Kingdom, indicating a need to take these differences into account when making cross-cultural comparisons. Anchoring vignettes offer a valid and feasible method for global data harmonization.

## Abbreviations

AV: anchoring vignette
BCHADS: Bangalore Child Health and Development Study
DIF: differential item functioning
EPDS: Edinburgh Postnatal Depression Scale
UKVA: UK Anchoring Vignette
WCHADS: Wirral Child Health and Development Study

The Edinburgh Postnatal Depression Scale (EPDS) (1) is a widely used screening questionnaire for postnatal depression in global research (2) and has been translated into over 60 languages (3). Substantial variation in how the measure functions across different cultural settings, including wide differences in validated cutpoints for identifying women at risk of postnatal depression (4, 5), indicates that cross-cultural comparisons using the tool should be interpreted carefully. This variation may help to explain some of the inconsistent findings observed in this area (6) and is likely to be driven, in part, by differential item functioning (DIF).

**Figure 1:**
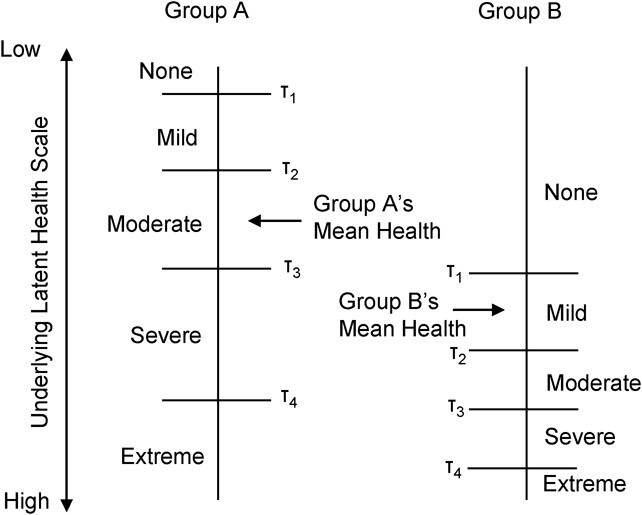
Illustrated example of differential item functioning. Individuals in groups A and B are asked to self-report their own level of health problems. How the average individual in each group divides the underlying latent trait into the 5 response categories is represented by τ_1_ to τ_4_. Differential item functioning is portrayed by variation in the placement of the thresholds across the two groups. Based on self-ratings, group A would be assumed to have more health problems, even though their actual level of health problems is lower.

DIF is present where response sets (e.g., never, sometimes, very often) have different meaning across cultures and individuals, implying that the same response to a given item does not represent the same level of the underlying latent trait. This can pose a significant challenge for self-assessments that depend upon an individual’s perception of the objective reality of their own true (latent) health and their subjective view of what it means to be above or below the given response thresholds (7). Problems arise when individual heterogeneity, resulting from the context or culture that different individuals live in and experience, causes those individuals to interpret and utilize these categories or thresholds in different ways. When this variation is systematic across groups or cultures, it can produce biased estimates of group differences (8) (Figure 1).

King et al. (9) proposed the anchoring vignette (AV) methodology as a solution to DIF. AVs are short narratives, normally 1 or 2 sentences long, that describe a hypothetical character and how that character is feeling or behaving in relation to a given construct (e.g., postnatal depression). These are translated into each target language so that the same vignettes can be shown to each group. Participants are typically first asked the self-assessment question(s) and then shown the vignettes and asked to rate the vignette characters using the same scale which they used to rate themselves. After careful translation, the vignette characters are assumed to exhibit the same level, or severity, of the concept being measured across groups. Because the true health of the vignette characters is “anchored” between groups, any systematic variation in ratings given between the groups can be attributed to DIF, also referred to as response style (7, 10, 11). By quantifying this variation, researchers can adjust group or individual responses to adhere to a common scale, thereby making the data directly comparable (12, 13). This adjustment is predicated on the assumptions of response consistency and vignette equivalence. Response consistency is the assumption that participants use the same subjective thresholds in rating vignettes as they use when rating themselves (7). Vignette equivalence is the assumption that “the level of the variable represented in any one vignette is perceived by all respondents in the same way and on the same unidimensional scale” (9, p. 194).

While this method has been successfully applied in various disciplines (9, 14–21), this approach is relatively novel in mental health research. Therefore, in the current study, we utilized a parametric modeling approach when applying the AV methodology to a multiple-item, variable response-set scale in the domain of mental health (14, 16, 18–20, 22–24). Uniquely, the UK perinatal cohort in our study was established prior to the Indian cohort, so a new auxiliary calibration sample was recruited in the United Kingdom, matched on key eligibility criteria, to generate a correction factor for ratings in the original sample. We believe this to be the first use of an auxiliary sample for such bias correction.

In this paper, we describe the development of a set of AVs for the EPDS and report the results of their application in samples in the United Kingdom and India with regard to 2 aims. The first was to investigate the utility and feasibility of the AV methodology in this context, and the second was to investigate whether and how DIF was affecting group comparisons of prevalence rates between India and the United Kingdom.

## METHODS

### Development

The key aim in developing AVs is to achieve response consistency and vignette equivalence (9). The EPDS contains 10 items, and each has a unique 4-item response set. Because DIF may act differently for each of the response sets, each item required a unique set of AVs. Ten sets of 6 vignettes were developed, with each set describing the item-specific symptom (e.g., crying, suicidal ideation) at a range of severity levels (from none to severe).

**Figure 2:**
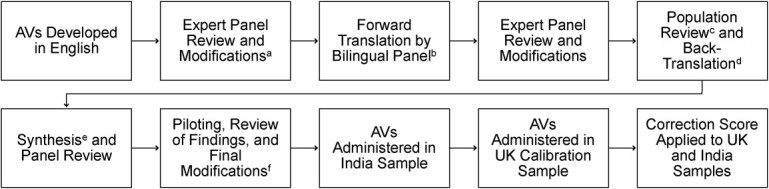
Development process for anchoring vignettes (AVs). ^a^Feedback provided regarding cross-cultural applicability, reading level, and the accuracy and range of symptom descriptions. ^b^Carried out by bilingual research assistants, with a focus on achieving functional equivalence. ^c^Carried out by a multinational team of academics and clinicians, focused on appropriateness of language used for the Kannada-speaking population and conceptual equivalence with English vignettes. ^d^Informal feedback gathered from members of the target population in India regarding vignette comprehension; vignettes back-translated by an independent bilingual clinician. ^e^Carried out by a multinational team of academics and clinicians, addressed comprehension issues identified in population review, and disparities in conceptual equivalence revealed by back-translation. ^f^Full details regarding the methodology, analysis, and results of the pilot test can be found in Web Appendix 1.

Figure 2 displays the many steps required for AV application.

#### Development in English.

Vignettes were written concisely using simple language and short sentences, describing the level of the depressive symptom for each corresponding EPDS item, with reference to specific behaviors and emotions and the frequency with which they occurred.

#### Translation into Kannada.

When translating the vignettes into Kannada, the local language in India, we followed guidance indicating that a functional approach to language translation improves vignette equivalence (25). Although the English set of vignettes was developed first, a flexible approach was retained throughout to allow for decentering and modification of both versions of vignettes simultaneously (26–28). The translation process is described in Figure 2.

#### Pilot testing.

The feasibility of administration of the EPDS in India, participant understanding, and indicators of vignette equivalence and response consistency were explored following recommendations in the literature (29). The vignettes were pilot-tested in a group of mothers (*n* = 32) who were part of the Bangalore Child Health and Development Study (BCHADS). Full details regarding the methodology, analysis, and results of the pilot test, including a ranking analysis, can be found in Web Appendix 1 (available at https://doi.org/10.1093/aje/kwad182) and Web Tables 1–11.

Findings indicated that participants demonstrated an ability to understand and complete the vignette task, that participants rated vignettes in the same way as they rated themselves, and that vignette content was relevant to participants’ lives. Some contextual limitations did exist, in terms of both the target population and the inherent characteristics of the EPDS, but in most cases minor procedural adjustments could be made to rectify these issues. Feedback regarding reporting behavior indicated that the response consistency assumption is likely to have been met, but there were several differences in the mean rank order given to the vignettes by participants in India and by a UK-based clinician.

An expert panel reviewed the pilot data and discussed changes in response to the findings. The Kannada vignettes were modified first and were then back-translated by a bilingual clinical psychologist who was blinded to the original vignettes and compared with their English counterparts.

### Application

The cross-cultural functioning and validity of the EPDS in 2 urban community samples in India and the United Kingdom was investigated using AVs. Data were drawn from 2 established cohorts and a new calibration sample recruited specifically for this project.

#### Bangalore Child Health and Development Study.

The BCHADS was a prospective epidemiologic cohort study of 909 pregnant women living in low-income areas of urban Bangalore, India. Women attending routine appointments were recruited from 3 community antenatal clinics between July 2014 and May 2016. This application used data from the follow-up assessments that took place 12 and 24 months after birth, including 549 participants for whom EPDS data were available and a nested subsample of 247 women who were eligible for assessment after the AVs had been added into the protocol.

#### Wirral Child Health and Development Study.

The Wirral Child Health and Development Study (WCHADS) was a prospective epidemiologic cohort study of 1,233 first-time mothers who had live singleton births and were recruited when attending a routine 20-week scan at a National Health Service hospital antenatal clinic in Wirral, United Kingdom, between February 2007 and October 2008. Full details of the sampling strategy and study methodology have been published elsewhere (30). This application used data from the follow-up assessment that took place 12 months after birth (*n* = 828).

#### UK Anchoring Vignette Study.

The UK Anchoring Vignette (UKAV) Study was an online cross-sectional study with participants recruited via social media and through Prolific Academic Ltd. (London, United Kingdom; www.prolific.co) between January 2020 and March 2020. The cohort consisted of 226 mothers who were living in the United Kingdom, were aged 18 years or older, could read or understand spoken English, and had at least 1 child aged 6–36 months. The Prolific function for representative samples was not utilized, as the additional eligibility criteria did not allow for a representative sample of sufficient size. Table 1 provides a comparison of key demographic factors between BCHADS, WCHADS, and the UKAV Study.

**Table 1:** Maternal Characteristics (%) in the Bangalore Child Health and Development Study (India, 2014–2016), the Wirral Child Health and Development Study (United Kingdom, 2007–2008), and the UK Anchoring Vignettes Study (United Kingdom, 2020)

	**Study**			
	**BCHADS**			
**Characteristic**	**Total** **(*n* = 549)**	**AV Subsample** **(*n* = 247)**	**WCHADS** **(*n* = 828)**	**UKAV Study** **(*n* = 226)**
Maternal age, years^a^	23.24 (3.56)	23.37 (3.57)	29.61 (5.68)	31.7 (5.15)
Employed	13.4	15.0	85.3	73.0
Secondary school education	70.1	64.0	71.5^b^	84.9^b^
Ethnicity				
White British	N/A	N/A	96.6	83.6
Other			3.4	16.4
Partnership status				
Married/cohabiting	100.0	100.0	81.6	91.6
Single	0.0	0.0	9.1	7.1
Other	0.0	0.0	9.3	1.9

Abbreviations: AV, anchoring vignette; BCHADS, Bangalore Child Health and Development Study; GCSE, General Certificate of Secondary Education; N/A, not applicable; UKAV, UK Anchoring Vignettes; WCHADS, Wirral Child Health and Development Study.

^a^ Values are expressed as mean (standard deviation).

^b^ Percentage of sample with 5 or more GCSEs.

### Measures

Maternal depression was assessed using the EPDS (1), a 10-item Likert scale self-report questionnaire. Each item addresses a distinct symptom of postnatal depression and is rated using a unique set of response items, scored 0–3, with a higher score indicating greater distress. The EPDS was self-administered in the UK samples and administered by research staff in the BCHADS. Respondents also completed AVs. Three EPDS items are reverse-scored (items 1, 2, and 4) and were reverse-coded for self-reports and AV ratings prior to analysis. Reichenheim et al. (31) reported, for a sample of Brazilian mothers of 5-month-olds, a strong first factor with loadings from 0.53 to 0.82.

### Procedure

#### Bangalore Child Health and Development Study.

At the 12-month assessment of the BCHADS, the EPDS was administered as part of a battery of maternal and child questionnaires. Due to low levels of literacy in the sample, the AVs were prerecorded, and women completed the ratings after listening to the audio recordings at 12 or 24 months. All researchers were provided with a standardized set of procedures and took part in group training and practice assessments.

Participants were asked to rate 2 vignettes for each of the 10 EPDS items. Participants were instructed to think of the vignette character as similar to themselves in age and background, to imagine themselves in the character’s position, and to pay attention to how the character was feeling and how long they had been feeling that way. Initially, an adaptive approach was used to automatically select vignettes based on the participant’s self-rating given for each EPDS item. However, low levels of EPDS reported symptoms meant that some AVs were rarely presented. The administration was altered to present 2 random vignettes from within each set.

#### Wirral Child Health and Development Study.

In the WCHADS, the EPDS was completed by mail (post) at 12 months.

#### UK Anchoring Vignette Study.

In the UKAV Study, the EPDS and the AVs were self-administered in the United Kingdom online using Qualtrics software (Qualtrics, Provo, Utah). Participants were first asked to complete a short demographic questionnaire and the EPDS in the standard format, followed by 2 random vignettes from each set. We used only their AV responses in this study. Instructions regarding the AV task mirrored those given to the BCHADS participants.

### Statistical analysis

**Figure 3:**
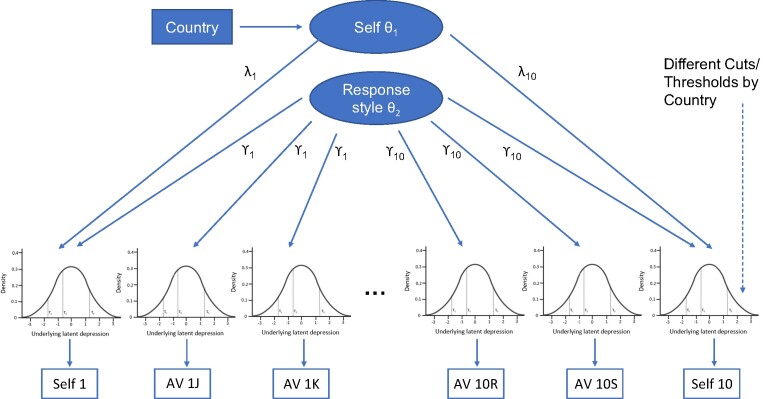
Schematic of the fitted anchoring vignette (AV)-adjusted model utilized to adjust group means for response style detected by the vignettes. Note that the 6 normal distribution figures are examples of possible threshold distribution and do not reflect real data.

Following both formal and informal checks that the AVs adequately met assumptions, for the main analyses we adopted a parametric modeling approach similar to that of Bolt et al. (32). Figure 3 shows a schematic of the fitted probit graded response model with 2 orthogonal factors estimated in Stata 17.0 (StataCorp LLC, College Station, Texas) using the “gsem” command, as shown in Web Appendix 2. The response style factor contributed to a notionally continuous normally distributed response for both AVs and self-reporting, which was then transformed by 3 thresholds into the ordered 4-category observed responses. The thresholds differed from item to item and between countries but were shared by both self-reporting and AVs. The self-report depression factor did not contribute to the AV responses, but in the absence of the AVs the self-report factor could not be distinguished from response style. We assumed that response style and the self-report factors were uncorrelated and that the factor loadings λ and γ were common across countries. Self-report depression in India was taken as the reference mean with the United Kingdom estimated relative to it, with response-style bias accounted for through differing thresholds. There was no assumption that the bias was in the same direction along the length of the item scale or that response style adhered to a specific a priori pattern (e.g., extreme, midpoint, acquiescence).

With estimation by maximum likelihood, the model has been shown to recover unbiased estimates in the presence of data missing at random (33). Confirmed by simulation, this includes situations where AVs are chosen randomly or adaptively dependent upon the participant’s self-report, and also where AVs are responded to by a different sample from those self-reporting, provided they are drawn from the same population (34).

### Ethics approval

Ethical approval for phases 1–5 of BCHADS was granted by the Institutional Ethics Committee of the Indian National Institute of Mental Health and Neuro-Sciences. Ethical approval for phases 6–9 was granted by the National Institute of Mental Health and Neuro-Sciences Institutional Ethics Committee and by the University of Liverpool Research Ethics Committee.

Ethical approval for WCHADS was granted by the Cheshire North and West Research Ethics Committee.

Ethical approval for the UKAV Study and an amendment to the study protocol was granted by the University of Liverpool Research Ethics Committee.

## RESULTS

### Descriptive results

**Figure 4:**
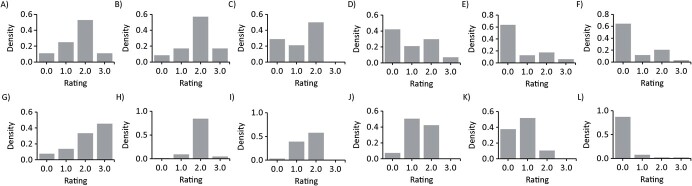
Distribution of Indian (A–F) and United Kingdom (G–L) anchoring vignette (AV) ratings for Edinburgh Postnatal Depression Scale item 1 (“I have been able to laugh and see the funny side of things”). A) Indian ratings of AV 1A; B) Indian ratings of AV 1B; C) Indian ratings of AV 1C; D) Indian ratings of AV 1D; E) Indian ratings of AV 1E; F) Indian ratings of AV 1F; G) UK ratings of AV 1A; H) UK ratings of AV 1B; I) UK ratings of AV 1C; J) UK ratings of AV 1D; K) UK ratings of AV 1E; L) UK ratings of AV 1F.

**Figure 5:**
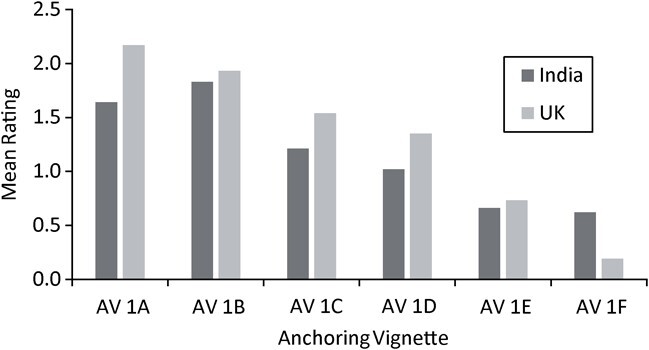
Comparison of United Kingdom and Indian mean anchoring vignette (AV) ratings for Edinburgh Postnatal Depression Scale item 1 (“I have been able to laugh and see the funny side of things”).

Figure 4 shows the distribution of UK and Indian participant ratings for the individual vignettes for EPDS item 1 (“I am able to laugh and see the funny side of things”) and shows that the vignettes were generally rated in the expected order. The vignettes generally showed a gradual shift from right to left, in line with decreasing severity. Combined mean ratings for each AV across countries are displayed for item 1 in Figure 5. There were order violations for items 3, 5, and 7–9, but all were quite small and were constrained to the more severe vignettes. For the remaining items, see Web Figures 1 and 2.

### Naive model

When estimated from self-assessments alone, with no AV-based bias correction, there was a medium-sized country difference in mean values for latent depression (*d* = 0.48, 95% confidence interval: 0.358, 0.599), with UK mothers reporting higher rates of postnatal depression than Indian mothers. Model estimates presented in Table 2 (naive model) show a strong factor with all loadings significant, but a substantial range from 0.27 (item 2) to 0.90 (item 8), indicating some items as contributing substantially more to the underlying depression construct than others. Thresholds varied between items, indicating that participants were applying unique thresholds to the response scales of different items and that some symptoms, such as item 3 (“I have blamed myself unnecessarily…”), were more common and associated with lower levels of depression than others.

**Table 2:** Summary Statistics From the Naive and AV-Adjusted Models^a^

	**EPDS Item**									
**Model Parameter**	**1**	**2**	**3**	**4**	**5**	**6**	**7**	**8**	**9**	**10**
*Naive Model* ^b^										
Shared coefficients^c^										
Latent depression loadings	0.54	0.27	0.69	0.74	0.76	0.69	0.84	0.90	0.83	0.71
Threshold 1	1.59	0.97	0.21	0.43	1.12	0.46	1.64	1.07	1.42	2.86
Threshold 2	2.24	1.40	1.26	1.39	1.96	1.58	2.70	2.99	2.80	3.37
Threshold 3	2.67	2.40	2.55	2.76	3.08	2.86	3.64	4.21	3.72	4.10
*AV-Adjusted Model* ^d^										
Shared coefficients^c^										
Latent depression loadings	0.68	0.48	0.64	0.72	0.77	0.67	0.81	0.86	0.83	0.74
Response style loadings	0.01	0.04	0.28	0.20	0.23	0.23	0.27	0.19	0.23	0.31
Vignette severity										
Vignette A	3.05	2.78	2.71	2.47	3.66	2.70	3.86	3.78	4.06	5.51
Vignette B	2.86	2.60	1.64	2.40	3.84	2.69	4.07	2.64	3.68	5.03
Vignette C	2.30	2.13	2.05	2.29	2.87	2.09	3.26	2.93	3.23	5.03
Vignette D	1.97	1.82	1.36	1.62	2.05	1.74	3.03	2.10	2.37	4.16
Vignette E	1.40	1.61	0.99	0.93	1.89	1.19	2.40	1.51	0.95	4.10
Vignette F	0.57	0.68	−0.25	−0.48	0.37	−0.70	0.13	−0.47	0.39	1.85
India										
Threshold 1	1.73	0.78	0.58	0.40	1.02	1.12	1.58	0.93	1.16	3.16
Threshold 2	2.19	1.11	1.53	1.33	2.03	1.62	2.52	2.46	2.11	4.14
Threshold 3	3.23	1.99	2.60	2.57	3.32	2.28	3.32	3.31	3.46	5.21
United Kingdom										
Threshold 1	1.17	1.01	−0.92	−0.41	0.28	−0.62	0.61	−0.37	0.45	2.43
Threshold 2	2.26	2.02	0.57	0.62	1.35	0.90	1.86	1.64	2.35	3.01
Threshold 3	3.85	2.89	2.30	2.34	3.07	2.73	3.41	3.27	3.77	4.20

Abbreviations: AV, anchoring vignette; CI, confidence interval; EPDS, Edinburgh Postnatal Depression Scale.

^a^ The naive model included the standardized factor loading of each item onto the latent depression factor, the response thresholds for each item, and the unadjusted mean difference in latent depression between countries. The AV model included the standardized shared factor loading for latent depression, the standardized shared factor loadings for within-country response style across all participants, the shared individual vignette coefficients, the unique item response threshold coefficients for India and the United Kingdom, and the adjusted mean difference in self-reported depression between the United Kingdom and India.

^b^ Overall difference in unadjusted latent depression mean values between the United Kingdom and India for the naive model = 0.47 (95% CI: 0.36, 0.59; *P* < 0.001).^c^ Coefficients shared between countries.

^c^ Coefficients shared between countries.

^d^ Overall difference in adjusted latent depression mean values between the United Kingdom and India for the AV-adjusted model = −0.25 (95% CI: −0.39, −0.10; *P* = 0.001).

### Allowing thresholds to differ by country

Table 2 (AV-adjusted model) displays the results from the model in which thresholds were allowed to differ by country as well as allowing random variation in individual response style bias within each country. The factor loadings for response style and latent depression factors are the same across groups by assumption. Differences in response style are evident in the differences in the thresholds between India and the United Kingdom. Additional individual differences in understanding are evident from the significance of the response style factor loadings, but the factor loadings are generally small, indicating a reasonably uniform understanding of the item questions and response set thresholds within each country, with items 7 and 10 being the most variably understood items. Allowing for country and individual response styles made for some modest differences in the depression factor loadings, with items appearing to perform a little more uniformly (standardized loadings ranging from 0.48 to 0.86) than in the naive model.

The position of each AV on the underlying depression scale was estimated relative to the mean self-report. Individual estimates (labeled vignettes A–F) are ordered corresponding to the descriptive mean ratings, indicating that the model estimated AV ratings as intended. Vignette ratings were almost all more severe than the mean self-report rating. Only the least severe vignettes for items 3, 5, 6, and 8 were rated as less severe than the mean self-report. The coefficients indicate that there was a relatively broad spread of ratings given to the vignettes, with the exception of item 10, for which AV coefficients for A–E are uniformly high.

After threshold adjustment the difference between countries in the mean depression factor was significant, but was now in the direction opposite (*d* = −0.25, 95% confidence interval: −0.391, −0.103) to that of the naive model, with UK respondents reporting lower levels of postnatal depression at 12 months after correction.

### Threshold/response style variation between the United Kingdom and India

**Figure 6:**
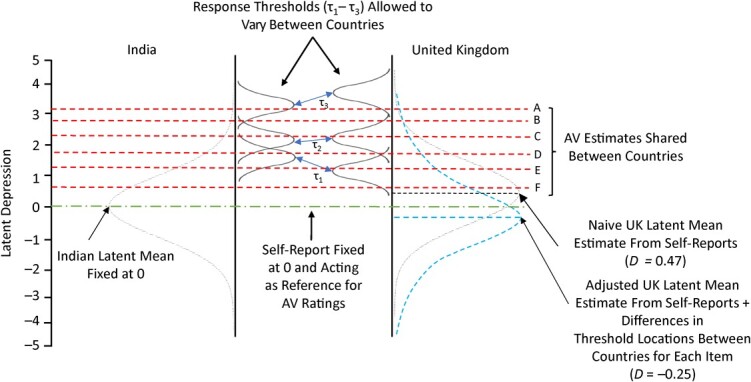
Anchoring vignette (AV)-adjusted model estimates for Edinburgh Postnatal Depression Scale item 1 (“I have been able to laugh and see the funny side of things”). The blue double-headed arrows (↔) show the difference in threshold locations estimated from differences in AV ratings between countries.

Of primary interest here are the different locations of the thresholds for each item between countries, most easily displayed graphically (Figure 6 and Web Figure 3). The response thresholds (τ_1_, τ_2_, τ_3_) are represented by approximate normal distribution curves in order to demonstrate that the likelihood of endorsing a given response changes based on the level of an individual’s underlying depression and response style. However, these curves are for illustration only and do not represent precise estimated distributions.

For the majority of items, model estimates indicate that UK participants used lower thresholds for τ_1_ and τ_2_, which represent the cutpoints between ratings of 0/1 and 1/2, and used similar or higher thresholds for τ_3_, which represents the cutpoint between ratings of 2/3. This indicates that, compared with Indian participants, UK participants required less severity to rate an item 1 or 2 but higher severity to rate an item 3. This dispersion of thresholds across the underlying distribution of the latent depression scale suggests that Indian mothers may have been relatively more likely to rate vignettes as 0 or 3, while UK mothers may have been relatively more likely to rate vignettes as 1 or 2.

Exceptions to the general pattern are item 2 (“I have looked forward with enjoyment to things”), in which all thresholds were higher for UK participants, and item 10 (“The thought of harming myself has occurred to me”), in which all thresholds were substantially lower for UK participants.

### How well do the AVs span the response thresholds?

On the whole, the vignettes appeared to divide up the spread of thresholds well but tended to be skewed toward the higher end of the distribution, markedly so for items 7 and 10. This might be considered desirable if correction for depression likely to be clinically significant is thought to be especially important.

### Example—EPDS item 1

To understand how the latent depression score measured by EPDS was adjusted using the AVs, we consider EPDS item 1 (“I have been able to laugh and see the funny side of things”) in detail.

Table 3 provides an example of the final AVs developed for item 1 in English and Kannada.

**Table 3:** Examples of Anchoring Vignettes Developed for Item 1^a^ of the Edinburgh Postnatal Depression Scale

**Anchoring** **Vignette**	**English Final Version**	**Kannada Final Version**	**English Back-Translation**
A	Jane is usually very cheerful but recently she has not been feeling like herself. Situations that normally wouldn’t bother her have been putting her in a bad mood. She used to laugh every day, but she can’t remember finding anything funny in the last week.	Lakshmi saadharanavaagi thumbaa khushiyaagi irutthaare. Aadhare munchinanthe anisuthilla. Maamuliyaagi avarige thondhare kodadha sandharbhagalu, eega avarannu ketta manasthithige tharutthidhe. Avaru dinaa nagunagutthaa irutthiddaru. Aadhare, kaledha ondhu vaaradindha avarige yenu thamashe thandhu koduva haage jnyaapaka barutthilla.	Lakshmi will usually be very happy. But she’s not feeling like before. The contexts/situations which usually won’t bother her are creating bad moods. She used [to] be cheerful every day. But from past one week, she can’t remember things which give her joy (she can’t remember any that will bring her fun).
B	Emily is usually very happy but work has been getting her down recently and she has been much more serious than usual. In the last week she has probably only laughed once.	Sameena saamaanyavaagi bahala santhoshadinda iruttare. Adare itthichege eladarinda kuggiddale haagu ghambeeravaagiddale. Kaleda ondu vaaradalli bahusha avaru onde baari nakkirabahudu.	Sameena will usually be very happy. But she is in low spirits because of day-to-day challenges and is very serious now. Probably, she must have laughed only once this past week.
C	Karen has always been a happy and positive person, but recently she has been feeling down about life. She would normally joke around and laugh with her friends every day, but she has only laughed a couple of times in the last week.	Meena yaavagalu santhoshavaagi iruttaare. Aadhare itthichege avarige jeevanadalli kuggutthiruvanthe anubhava aagutthide. Avaru saamanyavaagi thamaashe maaduttha, yellarondhige prathidina nagunaguthaa iddharu, aadhare kaledha vaaradalli avaru kevala vondheradu baari nakkiddhaare.	Meena is happy all the time. But she’s feeling low in life now a day. She usually used to make fun, used to be cheerful with everyone every day. But she has laughed/smiled one or two times in the last week.
D	Helen has been able to enjoy time with friends and family 3–4 days a week. The other half of the week she feels very low and barely able to force a smile.	Sitage thaanu munche iruvantheye anisuthade, haagu varadalli 3 athava 4 dinagalu, snehitharodane-kutumba davarodane anandisalu saadhyavaagutide. Ulida dinagalu avalu dukhadinda iddalu mattu nagalu saha kashta vagithu.	Sita feels that she has been the same as ever before, and 3 or 4 times in a week, she can enjoy the time with the friends and the family members. She was sad and had difficulty even to smile rest of the days.
E	Nicky has been enjoying life just as she usually does for most of the week. There have been a couple of times where she has struggled to cope when things haven’t gone to plan but otherwise she has been able to laugh off any difficulties.	Sheela vaaradalli hechhu dinagalu santhoshavagi iddaare. Andhukondanthe kelasagalu nadeyadiddaaga adannu nibhayisikondu hogalu kelavomme kashta vaagutthittu. Adannu bittare kashtadallu avalu nagalu sadyavaayithu.	Sheela is happy most of the days in a week. At times had difficulty to manage when the work didn’t happen in the expected way. Apart from that she could smile even in tough times.
F	Louise is normally very happy in life and this week has been no different. While she has been busy with different jobs that needed doing, she has been able to stop and spend some time laughing and having fun with her children 3 or 4 times a day.	Shwetha saamaanyavaagi santoshavaagi iruthare. Ee vaara kooda haage iddaru. Avaru maada bekaagiruva halavaaru kelasa karyagalunnu maadutta, kelavu samayavannu makkala jothege 3 rinda 4 baari moju mastiyalli kaleyuvaru.	Shwetha will usually be happy. She was feeling the same this week also. Along with daily chores which she is supposed to do, she spends time to have fun with children 3 or 4 times a day.

Abbreviations: AV, anchoring vignette; EPDS, Edinburgh Postnatal Depression Scale.

^a^ EPDS item 1: “I have been able to laugh and see the funny side of things.” Response options—“as much as I always could” (0); “not quite so much now” (1); “definitely not so much now” (2); “not at all” (3). AV response options—“as much as she always could” (0); “not quite so much now” (1); “definitely not so much now” (2); “not at all” (3). The number in parentheses is the value assigned to each response in the analyses.

Table 4 shows the mean ratings from the Indian and UK samples for each of the item 1 vignettes. Mean ratings were calculated from the values assigned to each of the response options, with a score of 0 for the option representing the lowest rating of depression (“As much as I always could”) and a score of 3 for the option representing the highest rating of depression (“Not at all”).

**Table 4:** Mean Anchoring Vignette Ratings and Corresponding Rankings (6 = Most Severe, 1 = Least Severe) for the Indian and UK Samples

	**Pilot Test**				**Empirical Application**			
	**India**		**United Kingdom**		**India**		**United Kingdom**	
**AnchoringVignette**	**Mean**	**Rank**	**Mean**	**Rank**	**Mean**	**Rank**	**Mean**	**Rank**
A (most severe)	1.50	5	N/A^a^	6	1.64	5	2.17	6
B	1.40	4	N/A	5	1.83	6	1.93	5
C	1.56	6	N/A	4	1.21	4	1.54	4
D	0.74	2	N/A	3	1.02	3	1.35	3
E	0.83	3	N/A	2	0.66	2	0.73	2
F (least severe)	0.25	1	N/A	1	0.62	1	0.19	1
Range	1.31		N/A		0.99		1.29	

Abbreviation: N/A, not applicable.

^a^ Pilot rankings in the United Kingdom were provided by a clinical psychologist with extensive experience working in a clinical and research setting with the target population. No mean ratings are available.

In the pilot phase, the mean ratings of vignettes 1A, 1B, and 1C were similar in the Indian sample, suggesting that the vignettes were not sufficiently distinct. AVs 1D and 1E were switched in relation to the expected rank order. Modifications were made to the text at this point to rectify ranking issues and to address participant feedback regarding difficulties in understanding the AVs (Web Appendix 1).

At the empirical stage, participants from both samples typically rated the AVs in the expected order. The exception to this is that the Indian sample rated vignette 1B at a higher level than vignette 1A. UK participants rated vignettes 1A–1E as representing higher levels of latent depression than the Indian sample, whereas vignette F was rated as representing a higher level of latent depression by the Indian sample. The range of ratings indicates that those of the Indian sample were typically compressed toward the middle of the scale, while UK participants used a wider range of responses.

Figure 6 illustrates how different response thresholds were estimated for the Indian and UK samples based on the AV ratings. A lower τ_1_ estimate for UK participants indicates that they had less demanding requirements for what constitutes “not quite so much.” A similar τ_2_ for UK and Indian participants indicates that they had similar requirements for what constitutes “definitely not so much.” A higher τ_3_ for UK participants indicates that they had more demanding requirements for what constitutes “no, not at all.” This pattern is broadly similar to the other AV sets, although τ_2_ was more often lower for UK participants than for Indian participants.

A higher τ_3_ indicates that UK mothers are less likely to give a rating of 3 to the AVs than Indian mothers. Intuitively, this might suggest that Indian mothers are more likely to give more severe ratings overall, which is not observed in the mean ratings. One reason for this may be the dispersion of the lower thresholds. As can be seen from Figure 6, the Indian τ_2_ and τ_1_ thresholds were typically higher than the corresponding UK thresholds, meaning that a larger proportion of the underlying latent depression scale fell below these lower thresholds in India. Consequently, the likelihood of Indian participants’ using these lower ratings was higher.

## DISCUSSION

To our knowledge, this is the first study using AVs that attempted to correct for reporting bias in postnatal depression when comparing 2 cohorts in the United Kingdom and India and the first study of any kind to use AVs collected in an auxiliary sample to perform such a correction, an approach that could substantially widen the scope of their application. The comparison of a naive, unadjusted model and an AV model that adjusted group means based on differences in the location of implicit cutpoints on the EPDS response scale gave entirely opposite findings. In the naive model, UK mothers were estimated as having higher levels of depression than Indian mothers. Following adjustment for response style, these positions were reversed, and the estimated level of depression was lower in UK mothers than in Indian mothers.

### What do the models tell us about differences in response style between India and the United Kingdom?

These results are indicative of a tendency for UK mothers with a particular level of depression to rate their own depressive symptoms more severely than equally depressed Indian mothers. Importantly, close examination of the thresholds used for dividing response categories shows that these differed between countries from one cutpoint to another and from one item to another. The dominant pattern is for lower levels of the latent trait to be rated more severely by UK mothers, but for UK mothers to require similar or higher levels of the latent trait to rate symptoms at the highest level.

### Are the adjusted latent mean values more accurate?

Because there is no objective indicator of postnatal depression, model validity was considered in relation to prevalence estimates in the existing literature. Published meta-analyses drawing from studies that have used a combination of clinical assessments and symptom questionnaires have consistently found higher rates of postnatal depression and other common mental disorders in low- and middle-income countries, including India (35–38), clearly supporting the direction of the adjustment in the current study.

### What is driving DIF between the UK and India?

King et al. (9) explain that DIF is likely rooted in different expectations regarding a specified construct. Our results indicate that UK participants generally have higher expectations regarding postnatal mental health, meaning that less severe symptoms are required for them to endorse higher ratings of depression in themselves, consistent with findings from previous studies (16, 19).

Differences in health expectations may be due to different levels of education or awareness regarding different aspects of health care (39), particularly perinatal mental health (35, 37). This position is supported by within-country AV studies which have found that education level is a significant predictor of DIF in a number of different settings (25, 40), in low- and middle-income countries (19, 41, 42), and specifically within India (10).

Alternatively, increased levels of socioeconomic adversity may be contributing to underreporting of symptoms in the Indian sample. Living in a resource-constrained setting increases the likelihood of being exposed to multiple adversities, particularly for women (43, 44). It is possible that this may result in lowered expectations regarding cognitive and emotional symptoms of postnatal depression, or in mothers in India appraising depression symptoms differently than those in high-income settings. It has been noted previously that questions addressing symptoms experienced during a specific time period (e.g., “in the past 7 days”) may be insensitive for women experiencing chronic adversity, since their experience of low mood may not vary from week to week (45).

### Limitations

Although it consists of only 10 items, the EPDS has 10 distinct response scales, some of which are complex. This meant that each item required a distinct set of AVs corresponding to the item content, and that some of the vignettes were relatively long and complex. Following translation into Kannada, the length of many vignettes grew substantially, adding considerably to the administration time and cognitive load placed on participants in India. A full pilot test of the AVs, including cognitive interviewing, was only carried out in India due to sample availability. In the United Kingdom, AVs were reviewed by a clinical psychologist with extensive clinical and research experience with the target population. There were also some differences in the administration of the EPDS and AVs between the United Kingdom and India. However, the impact of this is expected to have been small, as the mode of delivery for the EPDS and AVs was consistent within samples.

Our AV samples were relatively small, and in the case of UK participants (WCHADS vs. UKAV) they were drawn from a similar but not identical population. Additionally, a number of the mothers in the BCHADS sample did not understand spoken Kannada to a level sufficient to be included in the AV assessment. The BCHADS AV sample consisted of a relatively low-income urban population. As a result, these findings may not generalize to other populations in India, either in rural settings or in other states, which vary widely in terms of language, cultural practices, and sociodemographic factors (10).

### Strengths

To our knowledge, this is the first study to have used the AV method with a multiple-item and multiple–response-set scale in the field of maternal mental health, and it has shown the approach to be viable in this context. A multiple-item scale like the EPDS breaks down a broad construct into concrete symptoms thought to be universally experienced, potentially improving vignette equivalence. A robust and methodical approach was applied in the development, translation, and administration of the vignettes, with great care given to applying the knowledge and guidance available in the existing literature, increasing confidence in measurement assumptions.

### Implications and future directions for research

Our findings are consistent with previous within-country and cross-cultural AV studies that have found evidence of significant DIF which has dramatically affected comparisons between different groups in health-related research (10, 14–16, 18, 19, 25, 40–42, 46, 47) and other fields (9, 20, 21, 48).

The consistency of this evidence adds weight to the argument for data calibration between cultural groups. Without AV adjustment, any direct comparison between the two samples would likely have led to misleading conclusions. While the EPDS may be a valid predictor of important outcomes within India, the AV findings imply that cross-cultural comparison of raw scores on the EPDS should be used with caution.

### Conclusion

The implications of the current findings are potentially 2-fold. Firstly, the AV methodology is feasible and useful with the EPDS in this context. Secondly, differences in response style that indicate Indian participants are underreporting depressive symptoms relative to their UK counterparts confirm that direct comparisons between these two populations should not be taken at face value. Assuming that any systematic underreporting of symptoms would not be restricted to just postnatal depression, more work is urgently needed to understand the true burden of the whole range of perinatal mental health disorders in low- and middle-income country settings.

## Supplementary Material

Web_Material_kwad182
