# Supplementary material for: Experience and Reporting of Postnatal Depression Across Cultures: A Comparison Using Anchoring Vignettes of Mothers in the United Kingdom and India
Source: Am J Epidemiol. 2023 Sep 1;193(1):214–26. doi: 10.1093/aje/kwad182 (PMC10773478; doi:10.1093/aje/kwad182)
Supplement: Web_Material_kwad182 [file web_material_kwad182.pdf]

WEB MATERIAL

Experience and Reporting of Postnatal Depression Across Cultures: A Comparison Using Anchoring Vignettes of Mothers in the United Kingdom and India

Matthew Bluett-Duncan, Andrew Pickles, Prabha S. Chandra, Jonathan Hill, M. Thomas Kishore, Veena Satyanarayana, and Helen Sharp

Table of Contents

- Web Appendix 1 — Pp. 2–12
- Web Table 1 — P. 12
- Web Table 2 — P. 12
- Web Table 3 — P. 13
- Web Table 4 — P. 13
- Web Table 5 — P. 13
- Web Table 6 — P. 13
- Web Table 7 — P. 14
- Web Table 8 — P. 14
- Web Table 9 — P. 14
- Web Table 10 — P. 14
- Web Table 11 — P. 15
- Web Appendix 2 — Pp. 16–48
- Web Figure 1 — Pp. 49–53
- Web Figure 2 — P. 54
- Web Figure 3 — P. 55

Web Appendix 1

Pilot

Aim of the pilot study

Pilot testing forms a key part of developing new anchoring vignettes and there were several key areas that were explored during this part of the study. Of primary concern was whether the overall methodological approach was feasible in India and whether participants were able understand the vignettes. Understanding can be split into two areas. The first relates to semantic understanding. Did the participants understand the words and phrases used in the vignette, and does the overall vignette make sense to them? The second area relates to severity. Did participants understand the severity of the vignettes in the way that was expected (i.e., the *a priori* ranking)? Another key consideration was whether the vignette assumptions held for the vignettes. While it was beyond the scope of this study to perform stringent tests of assumptions, it was possible to examine various indicators of whether the assumptions were likely to be being met. A key paper that influenced the approach in this pilot study was Au and Lorgelly (1), particularly with regards to the assessment of response consistency (RC) and participant experience of the task. As such, that study was used as a standard against which several of the indicators from this study were compared.

Research questions

1. Is the established anchoring vignette methodology a feasible and valid approach in India?
2. Do participants show semantic comprehension of the vignettes?
3. Are the vignette assumptions likely to be met in the current sample?

Method

Sample

The overall anchoring vignette assessment sat within the BCHADS cohort and was designed to be completed by a subset of participants. Of this subset, a convenience sample of Kannada-speaking mothers, who were also completing the wider battery of assessments, participated in the pilot study by completing the vignette assessment and providing additional feedback. A total of 32 mothers participated in the vignette assessment at this point. Data from the pilot vignettes ratings were not included in any final anchoring vignette analyses.

Ethics

Ethical approval for the full BCHADS protocol, which included the use of the anchoring vignette methodology within the BCHADS sample, was given by NIMHANS and the University of Liverpool.

Measures

Maternal depression

Maternal depression was assessed in each cohort using the Edinburgh Postnatal Depression Scale (EPDS) (2), a 10-item Likert scale designed to detect depression in the postnatal period. The EPDS was translated into the local language (Kannada) following World Health Organisation guidelines for measure translation.

Anchoring vignettes

The anchoring vignettes were developed as part of this study for specific use with the EPDS. See the manuscript for details.

Pilot feedback questionnaire

A questionnaire was developed to record feedback and provide additional data for evaluating the vignettes. This questionnaire was administered alongside the anchoring vignettes with provision being made for feedback to be given during the task and following the completion of the task, as described below.

Feasibility: Participants were asked to provide feedback on how they found the task overall by rating the vignettes on ease of understanding and the amount of concentration required, both on a scale of 1-10. The RAs administering the pilot were also instructed to observe and record how the mothers responded to the instructions.

Semantic Comprehension: Participants were instructed to highlight any words or phrases that they found difficult to understand at the outset of the questionnaire and were reminded and asked for feedback after each vignette. Any comprehension issues, including the number of times each vignette was listened to, were recorded and collated so that recurrent difficulties could be identified.

Response Consistency and Vignette Rating Behaviour: If instructions were not understood or effective, it would raise questions as to the feasibility of the AV method in this context (3). Where findings suggested that this was the case, consideration was given to whether it was possible to make modifications to the instructions to enhance their effectiveness, without deviating substantially from what has been established in the literature. While Au and Lorgelly (1) conducted semi-structured interviews with participants, that was beyond the scope of this pilot study. Instead, a short set of questions were designed to explore similar domains and completed by participants immediately following completion of the vignette task. This provided valuable insight into the thought processes involved in completing the vignette task and whether the likelihood that the response consistency assumption held. The response consistency assumption was explored by asking participants how much they agreed with the statement, "I rated my health on the same health scale

(or in the same way) as I rated the health of the story characters". Responses were recorded on a 5-point scale (strongly agree, somewhat agree, neither agree nor disagree, somewhat disagree, and strongly disagree). Participants were also asked to describe their thoughts when rating the health of the vignette characters. Finally, participants were asked whether they assumed the characters were the same age and background as themselves, and whether they imagined themselves in their position. If the answer was negative, they were prompted to explain why.

Procedure

Prior to starting the vignette assessment, researchers introduced and explained the task to the participants using a pre-written script. Specific instructions to enhance response consistency were read out at the start of the task and then repeated every three vignettes to ensure that they were not forgotten. Following the task introduction, participants completed a practice vignette.

The EPDS and AV assessment were presented on a tablet using the Qualtrics online survey platform. Due to low literacy levels the EPDS was researcher administrated. Each EPDS item was read out and participants were invited to select their response from the four response items. The AVs were pre-recorded and presented orally in a random order following the completion of the EPDS. Playback of the vignettes was carried out using headphones to ensure that mothers could hear them clearly.

A split-half approach to vignette selection was used, representing a part standard presentation approach and part adaptive approach, whereby Qualtrics was programmed to randomly present two sets of vignettes. Set A contained all the vignettes (6 per item) for EPDS items 1, 3, 5, 7, and 9 (n=30) and two adaptively selected vignettes for EPDS items 2, 4, 6, 8, and 10 (n=10). Set B contained the reverse. This ensured that (i) each vignette was presented at least 15 times while not extending the pilot such that it would place excessive demands on participants and (ii) each EPDS item received corresponding vignette ratings which theoretically represent all points in the response set.

Each participant was therefore presented with 40 vignettes in total. Each vignette was played once through the headphones. Following this, the RA would read out the response options and the vignette was played again so the participant could listen to it with the response options in mind. The RA would then re-read the options and the participant would select the response. All participants were encouraged to listen to each vignette at least twice and informed that they could listen to the vignette as many times beyond that as they wanted. Following each vignette, participants were asked if there was anything they struggled to understand or did not make sense. Any feedback was recorded by the RA. The number of times each vignette was played was also recorded.

Following the completion of the AV task, participants were asked a series of questions relating to the feasibility of the task and their thought-processes and approach to the task.

Analysis plan

Analysis used a combination of quantitative and qualitative approaches. Any quantitative analysis was carried out in SPSS 24. Analysis was performed in relation to each key area of interest as follows:

Task feasibility

Mean participant ratings for ease of understanding and the amount of concentration required were calculated and compared with Au and Lorgelly (1). RA feedback was also considered.

Vignette comprehension (semantic)

Participant feedback was collated along with the number of times the vignettes were played. As most vignettes were played at least twice as part of the standard procedure, negative feedback was assumed where vignettes have been played 3 or more times. To determine the most troublesome vignettes, the two sources of data were combined and any vignette with 3 or more items of negative feedback was examined more closely.

Response consistency and vignette rating behaviour

Responses to the question, "I rated my health on the same health scale (or in the same way) as I rated the health of the story characters" were split into 3 categories regarding the likelihood of RC: Likely (strongly agree), Possible (somewhat agree, neither agree nor disagree), and Unlikely (somewhat disagree, strongly disagree). The proportion of responses for each category was calculated and compared against the responses reported by Au and Lorgelly (1).

The proportion of respondents giving positive and negative responses to the items, "did you assume that they were of the same age and background as yourself?" and "did you imagine yourself in their position?" was calculated and compared against the findings from Au and Lorgelly (1). The reasons given for negative responses were also examined.

Qualitative participant feedback regarding participant thought processes when rating the vignettes was examined in relation to their response to the items described above.

Vignette equivalence & comprehension (severity).

VE and severity comprehension were explored using a variation of the standard rank-order analysis used by King et al. (4). If rank ordering was consistent between groups (e.g., countries) this was taken as evidence that the vignettes were evoking similar levels of the relevant construct in each population and the VE assumption was considered to be fulfilled.

Due to time constraints imposed by the timeline of the larger BCHADS study it was not feasible to pilot in both the UK and India simultaneously, meaning a direct comparison of rank-ordering was not possible. Instead, the mean rank-ordering of vignettes in the Kannada sample was compared against the rank-ordering of a UK-based clinical psychologist. This individual was an expert in the area of maternal mental health and had worked extensively with the target population. It was therefore considered that she could offer a perspective on the rank ordering that was both clinically relevant and representative of the target population.

First, the mean response for each vignette across participants was calculated. There were four response items to select from for each vignette. These were scored from 0 to 3, with 0 representing the least severity of depressive symptoms and 3 representing the highest. These mean scores were then converted to ranks for each set of 6 vignettes. The rank order in the current sample for each set was then compared against the *a priori* ranking order and the ranking order of a UK clinical psychologist.

Some inconsistency in rank order was expected so it was important to distinguish between levels of inconsistencies. Where a vignette was two or more ranks out of expected position it was deemed to be a major violation. Where a vignette was one rank out of expected position it was deemed to be a minor violation. Where ranking inconsistencies were found the vignettes were reviewed to determine why this was the case and appropriate modifications were made.

Results.

Feasibility.

The mean rating for ease of understanding was 7.64 (SD = 2.43) and the mean rating for concentration required was 6.13 (SD = 3.20). Comparatively, the participants from Au and Lorgelly (1) gave a mean rating of 8.3 out of 10 for ease and 7.4 out of 10 for concentration required.

Response consistency and vignette rating behaviour.

When asked how much they agreed with the statement, *"I rated my health on the same health scale (or in the same way) as I rated the health of the story characters",* 37% said they "Strongly Agreed", 56.3% said they "Somewhat" agreed, 3.1% said they "Neither Agreed Nor Disagreed" and 3.1% said they "Strongly Disagreed". Comparatively, Au and Lorgelly (1) found that 38% of participants strongly agreed with the same statement.

100% of participants stated that they assumed the vignette characters were of the same age and background as themselves when rating the vignettes. 84.4% of participants stated they imagined themselves in the vignette scenario.

When participants were asked to describe their thought processes when rating the vignettes, most responded that they felt there was lots of similarity between their own lives and the situations and emotions described in the vignettes.

"What happens in my house every day, also happens in their lives"

"My family stories are also very similar. I could imagine myself in their place."

Participants also reported imagining themselves in the place of the vignette character, trying to understand how difficult it was for characters, and thinking about what decisions they would make if they were in their place.

"While answering I imagine myself as a character from some stories."

"I was trying to think what decisions I would take if I was in their place."

"I was trying to understand how difficult it is for them."

Only 5 out of 32 participants indicated that they did not relate to the vignette characters or content.

Research assistant feedback.

The research assistants observed that a lot of the participants were not paying attention to the duration or frequency of the different emotions described in the vignettes, but rather were just looking for whether a feeling was there or not. In other words, if an emotion was mentioned, participants appeared to rate it without considering its longevity or intensity. This was first noted from observations that a number of participants were consistently rating vignettes as very severe when symptoms were mentioned but described as very mild or non-existent. Further inspection of the preliminary pilot data confirmed this pattern.

Research staff reported that it was difficult to ensure that participants were paying attention and listening properly to the vignettes due to the format in which they were presented. Participants would occasionally appear to disengage while listening to the vignettes.

Researchers reported that many of the participants did not want to listen to the vignettes twice when asked to and insisted that they had understood them the first time. This was supported by the records of how many times each vignette was played, with a substantial proportion of mothers only listening to each vignette once during the pilot.

Vignette comprehension.

Six of the 60 pilot vignettes received ≥ 3 negative feedback indicators (participant feedback and vignette play count) and were therefore implicated as troublesome vignettes (Web Table 1).

Vignette equivalence and ranking comparison

Comparisons between the *a priori* rank order, UK clinician rank order and pilot rank order are presented in Web Tables 2-11). Means, standard deviations and coefficient of variations are presented for each vignette. Ranking inconsistencies were distinguished as either major (2 or more ranks out of place) or minor (1 rank out of place)..

Post-pilot review and modifications.

Review of vignettes using rank-order analysis.

Following the completion of the pilot data collection and analysis, an expert panel reviewed the data and discussed any changes that needed to be made to the vignettes. The panel consisted of the author, a clinical psychologist, a professor of biostatistics and psychometrics and a postdoctoral research assistant. All members of the panel have extensive experience in cross-cultural research. Following the agreed changes that were made by the panel, the modified vignettes were reviewed by the PI of BCHADS.

Modifications were made by examining the text in relation to rank order comparisons and vignette severity mean ratings. Several key issues were identified that could be driving rank order violations in the pilot data. The panel aimed to address these issues in the following ways. Firstly, to make any modifications that would enhance the conceptual equivalence of the vignettes. Secondly, to ensure that vignettes provide sufficiently distinct representations of symptom severity. Thirdly, to remove any possible primacy effects in the way vignettes are written. Data from the rank-order comparison was also cross-referenced with participant feedback.

A guiding principle of the review process was that changes were only made where absolutely necessary. Changes were only to be made where there was a clear rationale and where they were likely to result in significant contributions to meeting the aims described previously. Following modifications to the Kannada vignettes, the English vignettes were reviewed and compared to the Kannada vignette back-translations to determine if any changes could be made to further enhance vignette equivalence.

Discussion

Summary of findings

One of the main aims of the pilot study was to explore the feasibility of the AV method in India. Findings suggested that participants generally understood what was required of them and were able to complete the task. Some contextual limitations do exist, but in most cases minor procedural adjustments could be made to rectify these issues. Participants also generally demonstrated good semantic understanding of the vignette content. Thus, the AV approach appears to be valid and feasible in this context. Feedback regarding reporting behaviour indicates that the RC assumption is likely to have been met but rank-order comparisons revealed a number of inconsistencies between the expected and actual mean rank order. Further examination revealed that this was due to a number of reasons, including isolated cases of poor conceptual equivalence. These findings highlighted problem areas of the text which could then be modified appropriately.

Feasibility and administration.

Ratings regarding ease and concentration given in the current study were comparable to those found by Au and Lorgelly (1). Although participants found the vignettes slightly less easy to understand, they also reported that slightly less concentration was required to evaluate the characters. Feasibility was further demonstrated by participant feedback that the situations described in the vignettes were relevant to their lives and that many of them had experienced similar emotions and scenarios. As the scenarios were devised by a UK resident and approved by various UK experts, it can be assumed that they are also relevant to the UK context, thereby indicating that it is possible to write vignettes that are relevant to both target populations.

Due to the low literacy rate of the BCHADS sample, vignettes in this study were pre-recorded and played to participants using headphones. This made it difficult for test administrators to know when a participant had disengaged. Another limitation is that it is difficult to retain the necessary information in this format, and a participant cannot glance back over the text for a reminder of the relevant details. To combat this, vignettes were written to be as short and concise as possible, thereby minimising cognitive load. Additionally, the intended procedure was that participants would listen to each vignette at least twice. However, feedback suggested that many of the participants grew frustrated when being asked to listen to the vignettes twice and stated they did not need to. Due to the length and the details included in the vignettes, it was highly unlikely that participants were recollecting them accurately after listening to them once. Extra training was given to the test administrators prior to the full AV assessment so that they were able to provide better reasoning for why participants should listen to each vignette twice. However, where participants still did not want

to listen twice, they were not made to do so. Therefore, this needs to be considered as a limitation of the AV method in this context.

An element that may have added to participant frustration or disengagement with the AV task was the length of the Kannada vignettes. All of the vignettes increased in length to some degree, some very significantly, when translated from English. Although every effort was made to make the vignettes as concise as possible, the nature of the EPDS and the requirements of the AV method restricted these efforts. In view of the difficulties caused by the length of the Kannada vignettes, it may have been wiser to sacrifice some of the contextual information. While this may have detracted from RC fulfilment, it may have increased participant engagement in the overall task and reduced violations of VE. This trade-off is something to consider for researchers using the AV method in this context in the future. Researchers should consider how vignettes might be elongated in the target language when developing the initial vignettes so that they can make a more informed decision regarding the optimal balance of context and vignette length.

Vignette assumptions.

Test administrators noted that participants did not seem to be paying attention to the frequency or duration of emotions and behaviours in the vignettes. Instead, participants were focusing on whether an emotion or behaviour was present or not. Without the frequency, there is a lot of room for individual interpretation, which may have led to violations of the VE assumption. As a result, it was decided to add a direct instruction to pay attention to the duration of the symptoms that characters experienced. This was inserted into the procedure part way through the pilot and researchers noted that participants were paying more attention to the frequency descriptors as a result.

The established procedures also have a major impact on RC. Both the content and the timing of the instructions given to participants have been carefully curated to increase the likelihood of meeting this assumption (3). Thus, if results indicate that the RC assumption is not being met, this could be indicative that the established procedure, developed in a western setting, does not function adequately in an Indian context. However, 37 % of participants stated that they “strongly” agreed with the statement that they rated the vignette character in the same way that they rated themselves, while 56.3% said they “somewhat” agreed. Au and Lorgelly (1) gained similar results. They interpreted these responses as indicating that it was either “likely” or “possible” that the RC assumption was met, respectively, and reported that RC was likely to have been met for 38% of participants. Further to this in the current study, 100% of participants indicated that they followed

instructions and considered the participant to be the same age and background as themselves, and 84.4% imagined themselves in the position of the character when rating the vignette.

These findings were supported by feedback from many participants that the vignettes contained situations and emotions that they had experienced in their lives. With this being the case, participants will have been more easily able to empathise with the vignette characters and put themselves in their position. In turn, this increased the likelihood that participants rated the characters in the same way they rated themselves. While stringent tests of RC were beyond the scope of this study, Knott et al. (3) followed all the same recommendations and found that the RC assumption was met. Thus, it is reasonable to interpret the current findings as being indicative that the vignettes are likely to meet the RC assumption, and that the established procedure is effective in this regard.

The vignettes themselves were then considered and evaluated in detail. Using a rank-order comparison and participant feedback to draw attention to any possible issues, a panel of experts examined the text and made any necessary modifications. Rank order violations were found to arise for several reasons. Firstly, there were some inconsistencies between the focus of the vignettes and the cues given in the corresponding EPDS item. For example, item 2 reads *"I have looked forward with enjoyment to things".* While the majority of vignettes in this set followed the cue in the item and focused on the frequency and intensity of the positive emotion, there were two vignettes that focused on the frequency and intensity of an opposite negative emotion. It is possible that this resulted in a primacy effect that inflated perceptions of the severity of the depressive symptom. This issue was noted in several vignettes and corrected. Secondly, several of the vignettes contained unclear representations of symptom severity, such that it may have been difficult for participants to distinguish between vignettes when selecting response items. Some minor modifications were therefore required to clarify the intensity of symptoms portrayed. Thirdly, several vignettes were not adequately conceptually equivalent. In most cases, this was due to translation issues that arose because a certain word or phrase had been translated incorrectly. For example, one vignette refers to "household responsibilities". This was meant to refer to practical jobs around the house, but when translated into Kannada, took on the meaning of looking after the family in a more holistic sense. In other cases, conceptual differences were driven by phrases that had the same meaning but may have carried different cultural weights. For example, a description of someone who "manages with difficulty" may appear less remarkable and severe to an Indian participant due to increased socioeconomic adversity. Finally, there was one instance where the UK clinician and pilot rank order both disagreed with the *a priori* rank order. In this case, the *a priori* rank order was altered.

This process of review and modification was a vital part of the wider vignette development process. Beyond enhancing conceptual equivalence, it was also important that the vignettes were distinct enough that they clearly represented discrete levels of the relevant depressive symptom. If vignettes were not sufficiently distinct they would not provide a clear or accurate indication of DIF. Following the changes that were made to the vignettes in the post-pilot phase, the expert panel was satisfied that this was the case.***Limitations.***

Although every effort was made to enhance the validity of the vignettes, there were certain exogenous factors that could not be addressed by vignette design. Two of these issues arose from the EPDS itself. The phrasing of the items is complex and the BCHADS cohort has experienced difficulties in understanding and responding to them. Every effort was made in the initial translation of the EPDS to phrase the items in a way that was easily comprehensible to the study population but participants were still experiencing some difficulties. The second issue is that some of the EPDS response sets are more complex than others. For example, the first response option for item 7 is, *"Yes, sometimes",* whereas the first response option for item 6 is, *"Yes, sometimes I haven't been coping as well as usual".* The option for item 6 clearly requires more concentration and places a heavier cognitive load on participants than the option for item 7. This increases the likelihood of variation in ratings for item 6 as participants face the dual challenge of interpreting the meaning of both the vignette and the response set. There were also some English words that did not have a Kannada equivalent.

Conclusion

The pilot provided an opportunity to further develop the vignettes by observing how participants understood and evaluated them. It also afforded a vital insight into how the AV method functioned in this context. Findings indicated that participants demonstrated an ability to understand and complete the vignette task. Results also showed that participants rated vignettes in the same way as themselves and that vignette content was relevant to participants' lives. There were some limitations introduced by the EPDS itself and by the use of the AV method in the contrasting UK and Indian contexts. Some of these limitations could be addressed through minor procedural modifications. Other limitations, such as the nature of the EPDS, were outside of the control of the research team but they did not critically undermine the approach. Overall, the AV method appears to function well in this context and, following appropriate adjustments, should be successful in meeting the aim of enhancing cross-cultural comparisons of postnatal depression using data drawn from the EPDS. The final set of anchoring vignettes were therefore deemed acceptable for use in this context.

Web Table 1

Vignettes with ≥ 3 items of indicators of poor comprehension

	Frequency of Indicators		
Vignette	Participant Feedback	Play Count ≥3	Total Indicators
10D	1	4	5
1E	2	2	4
1C	1	2	3
1D	3	0	3
2D	1	2	3
6F	2	1	3

Web Table 2

Ranking comparison of vignette ratings for EPDS Item 1: *"I have been able to laugh and see the funny side of things"*.

Vignette	A Priori Rank	UK Clinician Rank	Pilot Rank	Mean Rating (SD, CoV)
1A	6	6	5*	1.5 (0.96, 0.64)
1B	5	5	4*	1.4 (0.9, 0.65)
1C	4	4	6**	1.56 (0.83, 0.57)
1D	3	3	2*	0.74 (0.79, 1.10)
1E	2	2	3*	0.83 (0.92, 1.10)
1F	1	1	1	0.25 (0.48, 1.79)

Note: *Pilot rank inconsistent with a priori rank by 1 rank, ** Pilot rank inconsistent with a priori rank by ≥2 ranks.

Web Table 3

Ranking comparison of vignette ratings for EPDS Item 2: *"I have looked forward with enjoyment to things"*

Vignette	A Priori Rank	UK Clinician Rank	Pilot Rank	Mean Rating (SD, CoV)
2A	6	6	5*	1.94 (0.97, 0.5)
2B	5	5	3**	1.78 (1.15, 0.63)
2C	4	4	2**	1.67 (1.15, 0.71)
2D	3	3	6**	1.97 (1.04, 0.53)
2E	2	2	4**	1.89 (1.21, 0.65)
2F	1	1	1	1.25 (0.92, 0.77)

Note: *Pilot rank inconsistent with a priori rank by 1 rank, ** Pilot rank inconsistent with a priori rank by ≥2 ranks.

Web Table 4

Ranking comparison of vignette ratings for EPDS Item 3: *"I have blamed myself unnecessarily when things went wrong"*.

Vignette	A Priori Rank	UK Clinician Rank	Pilot Rank	Mean Rating (SD, CoV)
3A	6	6	5*	1.69 (0.96, 0.56)
3B	5	5	3**	1.33 (0.9, 0.63)
3C	4	4	6**	1.84 (1.05, 0.55)
3D	3	3	4*	1.36 (0.65, 0.46)
3E	2	2	2	1.22 (0.65, 0.57)

3F	1	1	1	1.06 (1.00, 1.00)

Note: *Pilot rank inconsistent with a priori rank by 1 rank, ** Pilot rank inconsistent with a priori rank by ≥2 ranks.

Web Table 5

Ranking comparison of vignette ratings for EPDS Item 4: *"I have been anxious or worried for no good reason".*

Vignette	A Priori Rank	UK Clinician Rank	Pilot Rank	Mean Rating (SD, CoV)
4A	6	6	5*	1.95 (0.88, 0.45)
4B	5	5	4*	1.65 (1.15, 0.71)
4C	4	4	6**	2.1 (0.85, 0.41)
4D	3	3	3	1.62 (0.79, 0.51)
4E	2	2	2	1.24 (1.14, 0.91)
4F	1	1	1	0.67 (0.99, 1.45)

Note: *Pilot rank inconsistent with a priori rank by 1 rank, ** Pilot rank inconsistent with a priori rank by ≥2 ranks.

Web Table 6

Ranking comparison of vignette ratings for EPDS Item 5: *"I have felt scared or panicky for no very good reason".*

Vignette	A Priori Rank	UK Clinician Rank	Pilot Rank	Mean Rating (SD, CoV)
5A	6	6	5*	1.88 (0.8, 0.43)
5B	5	5	6*	2.06 (1.04, 0.5)
5C	4	4	3*	1.56 (0.74, 0.5)
5D	3	3	2*	1.31 (0.56, 0.41)
5E	2	2	4**	1.62 (0.91, 0.56)
5F	1	1	1	0.67 (0.77, 1.15)

Note: *Pilot rank inconsistent with a priori rank by 1 rank, ** Pilot rank inconsistent with a priori rank by ≥2 ranks.

Web Table 7

Ranking comparison of vignette ratings for EPDS Item 6: *"Things have been getting on top of me".*

Vignette	A Priori Rank	UK Clinician Rank	Pilot Rank	Mean Rating (SD, CoV)
6A	6	6	6	1.94 (1.2, 0.62)
6B	5	5	4*	1.67 (1.28, 0.77)
6C	4	4	5*	1.83 (1.2, 0.65)
6D	3	3	2*	1.32 (1.14, 0.88)
6E	2	2	3*	1.48 (1.13, 0.76)
6F	1	1	1	0.58 (1.01, 1.76)

Note: *Pilot rank inconsistent with a priori rank by 1 rank, ** Pilot rank inconsistent with a priori rank by ≥2 ranks.

Web Table 8

Ranking comparison of vignette ratings for EPDS Item 7: *"I have been so unhappy that I have had difficulty sleeping".*

Vignette	A Priori Rank	UK Clinician Rank	Pilot Rank	Mean Rating (SD, CoV)
7A	6	5***	5*	2.19 (0.95, 0.42)
7B	5	6***	6*	2.39 (0.88, 0.36)
7C	4	4	4	2.11 (1.02, 0.47)
7D	3	3	3	2 (1.03, 0.51)
7E	2	2	2	1.63 (0.99, 0.59)
7F	1	1	1	0.44 (0.88, 1.86)

Note: *Pilot rank inconsistent with a priori rank by 1 rank, ** Pilot rank inconsistent with a priori rank by ≥2 ranks.
***UK clinician rank inconsistent with a priori rank.

Web Table 9

Ranking comparison of vignette ratings for EPDS Item 8: *"I have felt sad or miserable"*.

Vignette	A Priori Rank	UK Clinician Rank	Pilot Rank	Mean Rating (SD, CoV)
8A	6	6	6	1.94 (0.99, 0.51)
8B	5	5	2**	1.16 (0.97, 0.83)
8C	4	4	5*	1.6 (0.85, 0.59)
8D	3	3	4*	1.59 (0.81, 0.52)
8E	2	2	3*	1.24 (0.75, 0.58)
8F	1	1	1	0.38 (0.82, 2.11)

Note: *Pilot rank inconsistent with a priori rank by 1 rank, ** Pilot rank inconsistent with a priori rank by ≥2 ranks.

Web Table 10

Ranking comparison of vignette ratings for EPDS Item 9: *"I have been so unhappy I have been crying"*.

Vignette	A Priori Rank	UK Clinician Rank	Pilot Rank	Mean Rating (SD, CoV)
9A	6	6	6	2.07 (0.9, 0.43)
9B	5	5	4*	1.88 (0.86, 0.46)
9C	4	4	5*	2 (0.77, 0.4)
9D	3	3	3	1.73 (0.69, 0.43)
9E	2	2	2	1.48 (0.59, 0.43)
1F	1	1	1	0.25 (0.48, 1.79)

Note: *Pilot rank inconsistent with a priori rank by 1 rank, ** Pilot rank inconsistent with a priori rank by ≥2 ranks.

Web Table 11

Ranking comparison of vignette ratings for EPDS Item 10: *"The thought of harming myself has occurred to me"*.

Vignette	A Priori Rank	UK Clinician Rank	Pilot Rank	Mean Rating (SD, CoV)
10A	6	6	6	1.94 (0.96, 0.5)
10B	5	5	5	1.88 (0.98, 0.53)
10C	4	4	4	1.82 (0.86, 0.48)
10D	3	3	3	1.59 (0.9, 0.61)
10E	2	2	2	1.53 (0.79, 0.52)
10F	1	1	1	0.12 (0.34, 2.82)

Note: *Pilot rank inconsistent with a priori rank by 1 rank, ** Pilot rank inconsistent with a priori rank by ≥2 ranks.

Web Appendix 2

Stata Code

```
. summ

    Variable |        Obs        Mean    Std. dev.       Min        Max
-------------+---------------------------------------------------------
     studyid |          0
        mage |      1,635    1.918043    5.754516         -7         26
         v_1 |      1,389    1.182865    .5550088          1          4
         v_2 |      1,387    1.312185    .6847252          1          4
         v_3 |      1,385    1.817329    .9118615          1          4
-------------+---------------------------------------------------------
         v_4 |      1,387    1.766402    .9290542          1          4
         v_5 |      1,388    1.491354    .8297359          1          4
         v_6 |      1,389    1.658027    .8318944          1          4
         v_7 |      1,389    1.408927    .7824301          1          4
         v_8 |      1,384    1.626445    .8562104          1          4
-------------+---------------------------------------------------------
         v_9 |      1,383    1.435286    .7579573          1          4
        v_10 |      1,389    1.054716    .3151341          1          4
      av_1_1 |        102    2.980392    .9332196          1          4
      av_1_2 |        120         2.9    .5710082          1          4
      av_1_3 |        130    2.446154    .6826935          1          3
-------------+---------------------------------------------------------
      av_1_4 |        280    2.128571    .9027466          1          4
      av_1_5 |        243    1.679012     .878962          1          4
      av_1_6 |        123    1.308943    .7143733          1          4
      av_2_1 |        128    3.382813    .8972495          1          4
      av_2_2 |        133    3.263158    .8429272          1          4
-------------+---------------------------------------------------------
      av_2_3 |        123    2.829268    .9295453          1          4
      av_2_4 |        270    2.792593    1.002571          1          4
      av_2_5 |        210    2.561905    1.144354          1          4
      av_2_6 |        134    1.716418    1.121304          1          4
      av_3_1 |        130    3.515385    .7391029          1          4
-------------+---------------------------------------------------------
      av_3_2 |        134    2.947761    .6862469          1          4
      av_3_3 |        144    3.138889    .8074055          1          4
      av_3_4 |        251    2.569721    .7142265          1          4
      av_3_5 |        222    2.288288    .7537224          1          4
      av_3_6 |        117    1.649573    .8233263          1          4
-------------+---------------------------------------------------------
      av_4_1 |        143    3.412587    .7901566          1          4
      av_4_2 |        130    3.384615    .7194379          1          4
      av_4_3 |        146    3.287671    .7330513          1          4
      av_4_4 |        239      2.8159    .7500689          1          4
      av_4_5 |        201    2.313433    .7185184          1          4
-------------+---------------------------------------------------------
      av_4_6 |        138    1.434783    .7443855          1          4
      av_5_1 |        141    3.638298    .6684374          1          4
      av_5_2 |        117    3.700855    .6055057          1          4
      av_5_3 |        127     3.19685    .7457751          1          4
      av_5_4 |        252    2.615079    .6426498          1          4
-------------+---------------------------------------------------------
      av_5_5 |        235    2.480851    .7060902          1          4
      av_5_6 |        126    1.539683    .8452294          1          4
      av_6_1 |        127    3.401575    .9365577          1          4
      av_6_2 |        131    3.343511    .9010335          1          4
      av_6_3 |        135    3.059259    .7895111          1          4
```

av_6_4	244	2.668033	1.042533	1	4
av_6_5	243	2.271605	.9227924	1	4
av_6_6	118	1.40678	.730733	1	4
av_7_1	128	3.570313	.7496308	1	4
av_7_2	119	3.672269	.6523652	1	4
av_7_3	146	3.246575	.7101398	1	4

```
      av_7_4 |        261    3.049808    .9453377          1          4
      av_7_5 |        235    2.553191    .8271181          1          4
      av_7_6 |        109    1.311927    .8355221          1          4
      av_8_1 |        129     3.55814     .799482          1          4
-------------+---------------------------------------------------------
      av_8_2 |        128    2.960938    .8639986          1          4
      av_8_3 |        153    3.150327    .7049752          1          4
      av_8_4 |        248    2.504032    .7420182          1          4
      av_8_5 |        213    2.131455    .6810066          1          4
      av_8_6 |        127    1.299213    .6584609          1          4
-------------+---------------------------------------------------------
      av_9_1 |        139    3.553957    .7534718          1          4
      av_9_2 |        125       3.384    .7597113          1          4
      av_9_3 |        134    3.119403    .7155079          1          4
      av_9_4 |        249    2.614458    .7099181          1          4
      av_9_5 |        218    2.311927    .6747213          1          4
-------------+---------------------------------------------------------
      av_9_6 |        133    1.774436    .9096831          1          4
     av_10_1 |        111    3.756757    .5910886          1          4
     av_10_2 |        111    3.522523    .8511687          1          4
     av_10_3 |        116    3.560345    .7137018          1          4
     av_10_4 |        278    2.751799    1.005175          1          4
-------------+---------------------------------------------------------
     av_10_5 |        269    2.732342    .9710908          1          4
     av_10_6 |        113    1.300885    .6251422          1          4
          uk |      1,642    .6552984    .4754157          0          1

. gen ukmage=mage*uk
(7 missing values generated)

. foreach item in 1 2 3 4 5 6 7 8 9 10 {
  2. rename v_`item' av_`item'_0
  3. }

. egen newid=group(studyid)

. reshape long av_1_ av_2_ av_3_ av_4_ av_5_ av_6_ av_7_ av_8_ av_9_ av_10_ ,i(newid)
j(meas)

Data                               Wide   ->   Long
-----------------------------------------------------------------------------
Number of observations            1,642   ->   11,494
Number of variables                  75   ->   17
j variable (7 values)                     ->   meas
xij variables:
               av_1_0 av_1_1 ... av_1_6   ->   av_1_
               av_2_0 av_2_1 ... av_2_6   ->   av_2_
               av_3_0 av_3_1 ... av_3_6   ->   av_3_
               av_4_0 av_4_1 ... av_4_6   ->   av_4_
               av_5_0 av_5_1 ... av_5_6   ->   av_5_
               av_6_0 av_6_1 ... av_6_6   ->   av_6_
               av_7_0 av_7_1 ... av_7_6   ->   av_7_
               av_8_0 av_8_1 ... av_8_6   ->   av_8_
               av_9_0 av_9_1 ... av_9_6   ->   av_9_
            av_10_0 av_10_1 ... av_10_6   ->   av_10_
                        v_0 v_1 ... v_6   ->   v_
-----------------------------------------------------------------------------

. ****************************
. *Without vignette correction
. ****************************
. tab meas, gen(m)

       meas |      Freq.     Percent        Cum.
------------+-----------------------------------
          0 |      1,642       14.29       14.29
```

1	1,642	14.29	28.57

```
          2 |      1,642       14.29       42.86
          3 |      1,642       14.29       57.14
          4 |      1,642       14.29       71.43
          5 |      1,642       14.29       85.71
          6 |      1,642       14.29      100.00
------------+-----------------------------------
      Total |     11,494      100.00

. dis "Naive IRT Model without correction"
Naive IRT Model without correction

. gsem (TRAIT[newid] -> av_1_@a av_2_ av_3_ av_4_ av_5_ ///
                        av_6_ av_7_ av_8_ av_9_ av_10_ , oprobit) ///
>       (uk -> TRAIT[newid]) if meas==0 , var(e.TRAIT[newid]@1) iter(20)

Fitting fixed-effects model:…
Iteration 6:   log likelihood = -9829.4601
Iteration 7:   log likelihood = -9829.4601

Generalized structural equation model                 Number of obs = 1,390

Response: av_1_                                       Number of obs = 1,389
Family:   Ordinal
Link:     Probit

Response: av_2_                                       Number of obs = 1,387
Family:   Ordinal
Link:     Probit

Response: av_3_                                       Number of obs = 1,385
Family:   Ordinal
Link:     Probit

Response: av_4_                                       Number of obs = 1,387
Family:   Ordinal
Link:     Probit

Response: av_5_                                       Number of obs = 1,388
Family:   Ordinal
Link:     Probit

Response: av_6_                                       Number of obs = 1,389
Family:   Ordinal
Link:     Probit

Response: av_7_                                       Number of obs = 1,389
Family:   Ordinal
Link:     Probit

Response: av_8_                                       Number of obs = 1,384
Family:   Ordinal
Link:     Probit

Response: av_9_                                       Number of obs = 1,383
Family:   Ordinal
Link:     Probit

Response: av_10_                                      Number of obs = 1,389
Family:   Ordinal
Link:     Probit

Log likelihood = -9829.4601

 ( 1)  [/]var(e.TRAIT[newid]) = 1
------------------------------------------------------------------------------------------
```

```
                          | Coefficient  Std. err.      z    P>|z|     [95% conf. interval]
--------------------------+-------------------------------------------------------------------
```

av_1_						
TRAIT[newid]	.6459126	.0628372	10.28	0.000	.522754	.7690712
av_2_						
TRAIT[newid]	.2774003	.0428862	6.47	0.000	.193345	.3614557
av_3_						
TRAIT[newid]	.9583727	.0523897	18.29	0.000	.8556907	1.061055
av_4_						
TRAIT[newid]	1.110342	.0608891	18.24	0.000	.9910016	1.229683
av_5_						
TRAIT[newid]	1.166424	.0716083	16.29	0.000	1.026074	1.306774
av_6_						
TRAIT[newid]	.9601788	.0542005	17.72	0.000	.8539478	1.06641
av_7_						
TRAIT[newid]	1.534279	.0986237	15.56	0.000	1.34098	1.727577
av_8_						
TRAIT[newid]	2.04624	.1353585	15.12	0.000	1.780942	2.311538
av_9_						
TRAIT[newid]	1.520613	.0955834	15.91	0.000	1.333273	1.707953
av_10_						
TRAIT[newid]	1.021758	.1263187	8.09	0.000	.7741775	1.269338
TRAIT[newid]						
uk	.4783112	.0615576	7.77	0.000	.3576605	.5989619
/av_1_						
cut1	1.58605	.0769634			1.435204	1.736895
cut2	2.236599	.0952466			2.049919	2.423278
cut3	2.672114	.1154108			2.445913	2.898315
/av_2_						
cut1	.97071	.0461225			.8803115	1.061109
cut2	1.399006	.0529119			1.295301	1.502711
cut3	2.400788	.1000591			2.204675	2.5969
/av_3_						
cut1	.2055282	.0613268			.0853299	.3257265
cut2	1.25524	.0715157			1.115072	1.395408
cut3	2.54586	.1008764			2.348146	2.743574
/av_4_						
cut1	.4280157	.0688104			.2931497	.5628816
cut2	1.386042	.0798372			1.229564	1.54252
cut3	2.756435	.1125039			2.535932	2.976939
/av_5_						
cut1	1.119994	.0834424			.9564494	1.283538
cut2	1.954829	.0984521			1.761866	2.147792
cut3	3.082748	.1345047			2.819123	3.346372
/av_6_						
cut1	.4586596	.0635308			.3341415	.5831777
cut2	1.583828	.0773499			1.432225	1.735431
cut3	2.863249	.1132158			2.64135	3.085148
/av_7_						
cut1	1.639649	.1173612			1.409625	1.869673

```
       cut2 |    2.70308   .1439075                      2.421026    2.985133
       cut3 |   3.637491   .1782626                      3.288102    3.986879
```

```
-------------------------------------+--------------------------------------------------------------------------------
/av_8_               |
               cut1 |   1.066238   .1258856                          .8195065    1.312969
               cut2 |    2.99574   .1847865                          2.633565    3.357915
               cut3 |   4.206357    .232855                          3.749969    4.662744
-------------------------------------+--------------------------------------------------------------------------------
/av_9_               |
               cut1 |   1.415539   .1085119                          1.202859    1.628218
               cut2 |   2.797871   .1423997                          2.518773     3.07697
               cut3 |   3.717315   .1777658                            3.3689    4.065729
-------------------------------------+--------------------------------------------------------------------------------
/av_10_              |
               cut1 |   2.860342   .2011649                          2.466066    3.254618
               cut2 |   3.370464   .2283946                          2.922819    3.818109
               cut3 |   4.103438    .309174                          3.497468    4.709408
-------------------------------------+--------------------------------------------------------------------------------
 var(e.TRAIT[newid])|          1  (constrained)
----------------------------------------------------------------------------------------------------------------------

. *****************************************************
. *With correction model with mean shift of thresholds
. egen group2=group(uk)

. gsem (av_1_ <- c.m2 c.m3 c.m4 c.m5 c.m6 c.m7 BIAS[newid] , oprobit) ///
>      (av_2_ <- c.m2 c.m3 c.m4 c.m5 c.m6 c.m7 BIAS[newid] , oprobit) ///
>          (av_3_ <- c.m2  c.m3 c.m4 c.m5 c.m6 c.m7 BIAS[newid] , oprobit) ///
>          (av_4_ <- c.m2 c.m3 c.m4 c.m5 c.m6 c.m7 BIAS[newid] , oprobit) ///
>          (av_5_ <- c.m2 c.m3 c.m4 c.m5 c.m6 c.m7 BIAS[newid] , oprobit) ///
>          (av_6_ <- c.m2 c.m3 c.m4 c.m5 c.m6 c.m7 BIAS[newid] , oprobit) ///
>          (av_7_ <- c.m2 c.m3 c.m4 c.m5 c.m6 c.m7 BIAS[newid] , oprobit) ///
>          (av_8_ <- c.m2 c.m3 c.m4 c.m5 c.m6 c.m7 BIAS[newid] , oprobit) ///
>          (av_9_ <-  c.m2 c.m3 c.m4 c.m5 c.m6 c.m7 BIAS[newid] , oprobit) ///
>          (av_10_ <- c.m2 c.m3 c.m4 c.m5 c.m6 c.m7 BIAS[newid] , oprobit) ///
>          (uk -> BIAS[newid]) ,  var(e.BIAS[newid]@1) iter(10) intp(7) intmethod(mv)
. matrix c=e(b)
. gsem (av_1_ <- c.m2 c.m3 c.m4 c.m5 c.m6 c.m7 BIAS[newid]  , oprobit) ///
>      (av_2_ <- c.m2 c.m3 c.m4 c.m5 c.m6 c.m7 BIAS[newid]  , oprobit) ///
>          (av_3_ <- c.m2 c.m3 c.m4 c.m5 c.m6 c.m7 BIAS[newid]  , oprobit) ///
>          (av_4_ <- c.m2 c.m3 c.m4 c.m5 c.m6 c.m7 BIAS[newid]  , oprobit) ///
>          (av_5_ <- c.m2 c.m3 c.m4 c.m5 c.m6 c.m7 BIAS[newid]  , oprobit) ///
>          (av_6_ <- c.m2 c.m3 c.m4 c.m5 c.m6 c.m7 BIAS[newid]  , oprobit) ///
>          (av_7_ <- c.m2 c.m3 c.m4 c.m5 c.m6 c.m7 BIAS[newid]  , oprobit) ///
>          (av_8_ <- c.m2 c.m3 c.m4 c.m5 c.m6 c.m7 BIAS[newid]  , oprobit) ///
>          (av_9_ <- c.m2 c.m3 c.m4 c.m5 c.m6 c.m7 BIAS[newid]  , oprobit) ///
>          (av_10_ <-  c.m2 c.m3 c.m4 c.m5 c.m6 c.m7 BIAS[newid] , oprobit) ///
>          (uk -> BIAS[newid]) ,  var(e.BIAS[newid]@1) iter(10) intp(7) ///
           intmethod(mv) from(c)

. matrix c=e(b)

. dis "Full Mean-Shift Model"
Full Mean-Shift Model

. gsem (av_1_ <- c.m2 c.m3 c.m4 c.m5 c.m6 c.m7 BIAS[newid] ///
                (c.m1#c.TRAIT[newid] , init(1)) , oprobit) ///
>      (av_2_ <- c.m2 c.m3 c.m4 c.m5 c.m6 c.m7 BIAS[newid] ///
       (c.m1#c.TRAIT[newid] , init(1)) , oprobit) ///
>      (av_3_ <- c.m2 c.m3 c.m4 c.m5 c.m6 c.m7 BIAS[newid] ///
       (c.m1#c.TRAIT[newid] , init(1)) , oprobit) ///
>      (av_4_ <- c.m2 c.m3 c.m4 c.m5 c.m6 c.m7 BIAS[newid] ///
       (c.m1#c.TRAIT[newid] , init(1)) , oprobit) ///
>      (av_5_ <- c.m2 c.m3 c.m4 c.m5 c.m6 c.m7 BIAS[newid] ///
       (c.m1#c.TRAIT[newid] , init(1)) ,oprobit) ///
>      (av_6_ <- c.m2 c.m3 c.m4 c.m5 c.m6 c.m7 BIAS[newid] ///
       (c.m1#c.TRAIT[newid] , init(1)) , oprobit) ///
>      (av_7_ <- c.m2 c.m3 c.m4 c.m5 c.m6 c.m7 BIAS[newid] ///
```

```
(c.m1#c.TRAIT[newid] , init(1)) , oprobit) ///
```

```
>       (av_8_ <- c.m2 c.m3 c.m4 c.m5 c.m6 c.m7 BIAS[newid] ///
        (c.m1#c.TRAIT[newid] , init(1)) , oprobit) ///
>       (av_9_ <- c.m2 c.m3 c.m4 c.m5 c.m6 c.m7 BIAS[newid] ///
        (c.m1#c.TRAIT[newid] , init(1)) , oprobit) ///
>       (av_10_ <- c.m2 c.m3 c.m4 c.m5 c.m6 c.m7 BIAS[newid] ///
        (c.m1#c.TRAIT[newid] , init(1)) , oprobit) ///
>       (uk -> BIAS[newid]) ((uk , init(0)) -> TRAIT[newid]), var(e.TRAIT[newid]@1) ///
>           from(c) var(e.BIAS[newid]@1) iter(20) intp(11) intmethod(mv)

Refining starting values:

Grid node 0:   log likelihood = -20863.544

Fitting full model:…

Iteration 6:   log likelihood = -20325.008
Iteration 7:   log likelihood = -20325.008

Generalized structural equation model                    Number of obs = 4,006

Response: av_1_                                          Number of obs = 2,387
Family:   Ordinal
Link:     Probit

Response: av_2_                                          Number of obs = 2,385
Family:   Ordinal
Link:     Probit

Response: av_3_                                          Number of obs = 2,383
Family:   Ordinal
Link:     Probit

Response: av_4_                                          Number of obs = 2,384
Family:   Ordinal
Link:     Probit

Response: av_5_                                          Number of obs = 2,386
Family:   Ordinal
Link:     Probit

Response: av_6_                                          Number of obs = 2,387
Family:   Ordinal
Link:     Probit

Response: av_7_                                          Number of obs = 2,387
Family:   Ordinal
Link:     Probit

Response: av_8_                                          Number of obs = 2,382
Family:   Ordinal
Link:     Probit

Response: av_9_                                          Number of obs = 2,381
Family:   Ordinal
Link:     Probit

Response: av_10_                                         Number of obs = 2,387
Family:   Ordinal
Link:     Probit

Log likelihood = -20325.008

 ( 1)  [av_1_]c.m1#TRAIT[newid] = 1
 ( 2)  [/]var(e.BIAS[newid]) = 1
 ( 3)  [/]var(e.TRAIT[newid]) = 1
------------------------------------------------------------------------------------------------
```

```
             | Coefficient  Std. err.      z    P>|z|     [95% conf. interval]
```

av_1_						
m2	3.01407	.1325025	22.75	0.000	2.75437	3.27377
m3	2.825108	.1245287	22.69	0.000	2.581037	3.06918
m4	2.320983	.1180822	19.66	0.000	2.089546	2.552419
m5	2.01058	.0964121	20.85	0.000	1.821616	2.199544
m6	1.454599	.1021161	14.24	0.000	1.254455	1.654743
m7	.763447	.1418781	5.38	0.000	.4853709	1.041523
BIAS[newid]	.0328233	.0277147	1.18	0.236	-.0214966	.0871432
c.m1#TRAIT[newid]	1	(constrained)				
av_2_						
m2	2.641638	.1204207	21.94	0.000	2.405618	2.877658
m3	2.467205	.1154902	21.36	0.000	2.240849	2.693562
m4	1.975771	.1124387	17.57	0.000	1.755395	2.196146
m5	1.8264	.0849335	21.50	0.000	1.659934	1.992867
m6	1.61129	.091656	17.58	0.000	1.431647	1.790932
m7	.7464824	.1164283	6.41	0.000	.5182871	.9746777
BIAS[newid]	-.2124341	.025542	-8.32	0.000	-.2624956	-.1623727
c.m1#TRAIT[newid]	.4997646	.0508726	9.82	0.000	.4000561	.599473
av_3_						
m2	2.85545	.1375959	20.75	0.000	2.585767	3.125133
m3	1.753166	.1205427	14.54	0.000	1.516907	1.989425
m4	2.168497	.1222016	17.75	0.000	1.928986	2.408008
m5	1.509547	.0995189	15.17	0.000	1.314493	1.7046
m6	1.1389	.1006984	11.31	0.000	.9415346	1.336265
m7	-.0289611	.1248873	-0.23	0.817	-.2737358	.2158135
BIAS[newid]	.5134778	.0349789	14.68	0.000	.4449205	.5820351
c.m1#TRAIT[newid]	.9311203	.0538191	17.30	0.000	.8256367	1.036604
av_4_						
m2	2.582311	.1277053	20.22	0.000	2.332013	2.832609
m3	2.483177	.1299699	19.11	0.000	2.22844	2.737913
m4	2.389258	.1239788	19.27	0.000	2.146264	2.632251
m5	1.753761	.103179	17.00	0.000	1.551534	1.955988
m6	1.058472	.1034615	10.23	0.000	.855691	1.261253
m7	-.3023944	.1250771	-2.42	0.016	-.547541	-.0572478
BIAS[newid]	.3040569	.0286757	10.60	0.000	.2478536	.3602602
c.m1#TRAIT[newid]	1.121044	.0592641	18.92	0.000	1.004888	1.237199
av_5_						
m2	3.730746	.1540947	24.21	0.000	3.428726	4.032766
m3	3.889604	.1670579	23.28	0.000	3.562177	4.217032
m4	2.962255	.1441497	20.55	0.000	2.679726	3.244783
m5	2.197404	.1182429	18.58	0.000	1.965653	2.429156
m6	2.044447	.1186375	17.23	0.000	1.811922	2.276972
m7	.5538427	.1376652	4.02	0.000	.2840238	.8236616
BIAS[newid]	.3269621	.0318	10.28	0.000	.2646352	.389289
c.m1#TRAIT[newid]	1.353427	.076437	17.71	0.000	1.203613	1.503241
av_6_						
m2	2.500532	.1277893	19.57	0.000	2.250069	2.750994
m3	2.410069	.1244342	19.37	0.000	2.166182	2.653956
m4	1.965813	.115543	17.01	0.000	1.739353	2.192273

```
          m5 |   1.696486   .0963346    17.61   0.000     1.507674    1.885299
          m6 |   1.196187   .0934931    12.79   0.000     1.012944     1.37943
```

m7	-.353585	.1304797	-2.71	0.007	-.6093205	-.0978495
BIAS[newid]	.393069	.0296916	13.24	0.000	.3348746	.4512634
c.m1#TRAIT[newid]	.8026878	.0501249	16.01	0.000	.7044449	.9009308
av_7_						
m2	3.758372	.1688067	22.26	0.000	3.427517	4.089227
m3	3.960425	.1770283	22.37	0.000	3.613456	4.307394
m4	3.173773	.1538639	20.63	0.000	2.872205	3.47534
m5	3.077709	.1407863	21.86	0.000	2.801773	3.353645
m6	2.471017	.1371785	18.01	0.000	2.202152	2.739882
m7	.4101355	.1711244	2.40	0.017	.0747379	.7455331
BIAS[newid]	.3520549	.0345821	10.18	0.000	.2842753	.4198345
c.m1#TRAIT[newid]	1.575414	.0907102	17.37	0.000	1.397625	1.753203
av_8_						
m2	3.856335	.1780211	21.66	0.000	3.50742	4.20525
m3	2.752597	.1624138	16.95	0.000	2.434272	3.070923
m4	3.039774	.160292	18.96	0.000	2.725607	3.35394
m5	2.26008	.1435402	15.75	0.000	1.978746	2.541413
m6	1.657102	.1421242	11.66	0.000	1.378544	1.93566
m7	-.1581231	.1632981	-0.97	0.333	-.4781815	.1619354
BIAS[newid]	.3879332	.0331883	11.69	0.000	.3228852	.4529811
c.m1#TRAIT[newid]	1.939206	.0984655	19.69	0.000	1.746217	2.132195
av_9_						
m2	3.926493	.1630157	24.09	0.000	3.606988	4.245997
m3	3.56134	.1606013	22.18	0.000	3.246567	3.876113
m4	3.151968	.1535682	20.52	0.000	2.85098	3.452956
m5	2.531125	.134252	18.85	0.000	2.267996	2.794254
m6	2.135189	.1339484	15.94	0.000	1.872655	2.397723
m7	1.221013	.1445428	8.45	0.000	.9377143	1.504312
BIAS[newid]	.2017869	.0299934	6.73	0.000	.1430009	.2605729
c.m1#TRAIT[newid]	1.634098	.0889052	18.38	0.000	1.459847	1.808349
av_10_						
m2	5.871311	.2648492	22.17	0.000	5.352216	6.390405
m3	5.375104	.2496874	21.53	0.000	4.885725	5.864482
m4	5.376011	.2498542	21.52	0.000	4.886306	5.865717
m5	4.519555	.2265324	19.95	0.000	4.07556	4.963551
m6	4.481934	.22615	19.82	0.000	4.038688	4.92518
m7	2.134969	.2292649	9.31	0.000	1.685618	2.584319
BIAS[newid]	.5277749	.0448397	11.77	0.000	.4398906	.6156592
c.m1#TRAIT[newid]	1.363466	.1343339	10.15	0.000	1.100177	1.626756
BIAS[newid]						
uk	1.870513	.1180121	15.85	0.000	1.639213	2.101812
TRAIT[newid]						
uk	-.0498818	.0672602	-0.74	0.458	-.1817092	.0819457
/av_1_						
cut1	1.568206	.075003			1.421203	1.715209
cut2	2.312704	.0803502			2.15522	2.470187
cut3	3.585993	.0940194			3.401719	3.770268

```
/av_2_               |
              cut1 |   .7160051   .0544971                       .6091928    .8228174
```

```
              cut2 |   1.325107   .0579758                    1.211477    1.438738
              cut3 |   2.176237   .0658045                    2.047262    2.305211
-------------------+----------------------------------------------------------------
/av_3_             |
              cut1 |   .4404188   .0714235                    .3004314    .5804062
              cut2 |   1.690787     .08285                    1.528404     1.85317
              cut3 |   3.168085   .1014419                    2.969262    3.366907
-------------------+----------------------------------------------------------------
/av_4_             |
              cut1 |   .4118836   .0725929                    .2696042     .554163
              cut2 |   1.396836   .0791535                    1.241698    1.551974
              cut3 |   2.881953   .0945433                    2.696652    3.067255
-------------------+----------------------------------------------------------------
/av_5_             |
              cut1 |   1.126497    .092815                    .9445833    1.308412
              cut2 |   2.153888   .1034333                    1.951162    2.356613
              cut3 |   3.621973   .1223172                    3.382236     3.86171
-------------------+----------------------------------------------------------------
/av_6_             |
              cut1 |   .6798076   .0663984                    .5496691    .8099462
              cut2 |   1.663945   .0752846                     1.51639      1.8115
              cut3 |   2.817342   .0879541                    2.644956    2.989729
-------------------+----------------------------------------------------------------
/av_7_             |
              cut1 |    1.57463   .1134885                    1.352197    1.797064
              cut2 |   2.614946   .1277811                    2.364499    2.865392
              cut3 |   3.685354   .1398116                    3.411329     3.95938
-------------------+----------------------------------------------------------------
/av_8_             |
              cut1 |   .8616972   .1158211                     .634692    1.088702
              cut2 |   2.614232   .1352871                    2.349074     2.87939
              cut3 |    3.84663   .1489849                    3.554625    4.138635
-------------------+----------------------------------------------------------------
/av_9_             |
              cut1 |   1.177288    .107533                    .9665277    1.388049
              cut2 |   2.517424   .1216726                     2.27895    2.755898
              cut3 |   3.806933   .1349533                     3.54243    4.071437
-------------------+----------------------------------------------------------------
/av_10_            |
              cut1 |   3.603038   .2120282                     3.18747    4.018605
              cut2 |   4.433838   .2278751                    3.987211    4.880465
              cut3 |   5.545915   .2452751                    5.065184    6.026645
-------------------+----------------------------------------------------------------
  var(e.BIAS[newid])|          1  (constrained)
 var(e.TRAIT[newid])|          1  (constrained)
------------------------------------------------------------------------------------

. estat ic

Akaike's information criterion and Bayesian information criterion

-----------------------------------------------------------------------------
       Model |          N   ll(null)  ll(model)      df         AIC        BIC
-------------+---------------------------------------------------------------
           . |      4,006          .  -20325.01     111    40872.02   41570.82
-----------------------------------------------------------------------------
Note: BIC uses N = number of observations. See [R] BIC note.

. ************************
. ***Free threshold model
. ************************
. gsem (av_1_ <-  c.m2 c.m3 c.m4 c.m5 c.m6 c.m7 (BIAS[newid], init(0.1)) , oprobit) ///
>      (av_2_ <-  c.m2 c.m3 c.m4 c.m5 c.m6 c.m7 (BIAS[newid], init(0.1)) , oprobit) ///
>      (av_3_ <-  c.m2 c.m3 c.m4 c.m5 c.m6 c.m7 (BIAS[newid], init(0.1)) , oprobit) ///
>      (av_4_ <-  c.m2 c.m3 c.m4 c.m5 c.m6 c.m7 (BIAS[newid], init(0.1)) , oprobit) ///
>      (av_5_ <-  c.m2 c.m3 c.m4 c.m5 c.m6 c.m7 (BIAS[newid], init(0.1)) , oprobit) ///
```

```
>      (av_6_ <-  c.m2 c.m3 c.m4 c.m5 c.m6 c.m7 (BIAS[newid], init(0.1)) , oprobit) ///
```

```
>       (av_7_ <-  c.m2 c.m3 c.m4 c.m5 c.m6 c.m7 (BIAS[newid], init(0.1)) , oprobit) ///
>       (av_8_ <-  c.m2 c.m3 c.m4 c.m5 c.m6 c.m7 (BIAS[newid], init(0.1)) , oprobit) ///
>       (av_9_ <-  c.m2 c.m3 c.m4 c.m5 c.m6 c.m7 (BIAS[newid], init(0.1)) , oprobit) ///
>    (av_10_ <-  c.m2 c.m3 c.m4 c.m5 c.m6 c.m7 (BIAS[newid], init(0.1)) , oprobit) , ///
>           group(group2) mean(BIAS[newid]@0)  var(BIAS[newid]@1) ginvariant(load scale
coef) iter(10) intp(7)

. matrix z=e(b)

.
. dis "Full Free-Threshold Model"
Full Free-Threshold Model

. gsem (av_1_ <- c.m2 c.m3 c.m4 c.m5 c.m6 c.m7 BIAS[newid] ///
       (c.m1#c.TRAIT[newid] , init(1)) , oprobit) ///
>       (av_2_ <- c.m2 c.m3 c.m4 c.m5 c.m6 c.m7 BIAS[newid] ///
       (c.m1#c.TRAIT[newid] , init(1)) , oprobit) ///
>       (av_3_ <- c.m2 c.m3 c.m4 c.m5 c.m6 c.m7 BIAS[newid] ///
       (c.m1#c.TRAIT[newid] , init(1)) , oprobit) ///
>       (av_4_ <- c.m2 c.m3 c.m4 c.m5 c.m6 c.m7 BIAS[newid] ///
       (c.m1#c.TRAIT[newid] , init(1)) , oprobit) ///
>       (av_5_ <- c.m2 c.m3 c.m4 c.m5 c.m6 c.m7 BIAS[newid] ///
       (c.m1#c.TRAIT[newid] , init(1)) , oprobit) ///
>       (av_6_ <- c.m2 c.m3 c.m4 c.m5 c.m6 c.m7 BIAS[newid] ///
       (c.m1#c.TRAIT[newid] , init(1)) , oprobit) ///
>       (av_7_ <- c.m2 c.m3 c.m4 c.m5 c.m6 c.m7 BIAS[newid] ///
       (c.m1#c.TRAIT[newid] , init(1)) , oprobit) ///
>       (av_8_ <- c.m2 c.m3 c.m4 c.m5 c.m6 c.m7 BIAS[newid] ///
       (c.m1#c.TRAIT[newid] , init(1)) , oprobit) ///
>       (av_9_ <-  c.m2 c.m3 c.m4 c.m5 c.m6 c.m7 BIAS[newid] ///
       (c.m1#c.TRAIT[newid] , init(1)) , oprobit) ///
>       (av_10_ <-  c.m2 c.m3 c.m4 c.m5 c.m6 c.m7 BIAS[newid] ///
       (c.m1#c.TRAIT[newid] , init(1)) , oprobit) , ///
>      group(group2) var(1:BIAS[newid]@1) mean(1:BIAS[newid]@0) ///
       var(2:BIAS[newid]@1) mean(2:BIAS[newid]@0) ///
>      var(1:TRAIT[newid]@1)  var(2:TRAIT[newid]@1) ///
       mean(1:TRAIT[newid]@0) mean(2:TRAIT[newid])  ///
>      cov(1:BIAS[newid]*TRAIT[newid]@0) cov(2:BIAS[newid]*TRAIT[newid]@0) ///
       ginvariant(load scale coef)from(z) iter(30) intp(11)

Fitting fixed-effects model:


Iteration 10:  log likelihood = -19691.355
Iteration 11:  log likelihood = -19691.354

Generalized structural equation model                 Number of obs    = 4,006
Grouping variable: group2                             Number of groups =     2
Log likelihood = -19691.354

Group:    1                                              Number of obs = 1,694

Response: av_1_                                          Number of obs = 1,060
Family:   Ordinal
Link:     Probit

Response: av_2_                                          Number of obs = 1,057
Family:   Ordinal
Link:     Probit

Response: av_3_                                          Number of obs = 1,059
Family:   Ordinal
Link:     Probit

Response: av_4_                                          Number of obs = 1,060
Family:   Ordinal
```

Link: Probit

Response: av_5_ Number of obs = 1,060
Family: Ordinal
Link: Probit

Response: av_6_ Number of obs = 1,060
Family: Ordinal
Link: Probit

Response: av_7_ Number of obs = 1,060
Family: Ordinal
Link: Probit

Response: av_8_ Number of obs = 1,060
Family: Ordinal
Link: Probit

Response: av_9_ Number of obs = 1,058
Family: Ordinal
Link: Probit

Response: av_10_ Number of obs = 1,060
Family: Ordinal
Link: Probit

	Coefficient	Std. err.	z	P>|z|	[95% conf.	interval]
av_1_						
m2	3.045255	.146489	20.79	0.000	2.758142	3.332368
m3	2.863498	.1410171	20.31	0.000	2.58711	3.139887
m4	2.298565	.1327731	17.31	0.000	2.038334	2.558795
m5	1.967435	.1128217	17.44	0.000	1.746309	2.188562
m6	1.398545	.116863	11.97	0.000	1.169498	1.627593
m7	.5683581	.1491886	3.81	0.000	.2759537	.8607624
BIAS[newid]	-.0166117	.0430374	-0.39	0.700	-.1009635	.0677401
c.m1#TRAIT[newid]	.9309581	.0787886	11.82	0.000	.7765352	1.085381
av_2_						
m2	2.782966	.1219367	22.82	0.000	2.543974	3.021957
m3	2.59733	.1178091	22.05	0.000	2.366428	2.828231
m4	2.12574	.1146923	18.53	0.000	1.900947	2.350533
m5	1.823478	.0865834	21.06	0.000	1.653777	1.993178
m6	1.608219	.09298	17.30	0.000	1.425982	1.790457
m7	.6774317	.1166204	5.81	0.000	.4488598	.9060036
BIAS[newid]	-.0510329	.0390731	-1.31	0.192	-.1276148	.0255489
c.m1#TRAIT[newid]	.5517016	.0527336	10.46	0.000	.4483456	.6550575
av_3_						
m2	2.714612	.1330214	20.41	0.000	2.453895	2.975329
m3	1.644821	.1178975	13.95	0.000	1.413746	1.875896
m4	2.052563	.1186465	17.30	0.000	1.82002	2.285106
m5	1.361059	.0947411	14.37	0.000	1.17537	1.546748
m6	.9955193	.0964128	10.33	0.000	.8065536	1.184485
m7	-.2510729	.1221723	-2.06	0.040	-.4905262	-.0116195
BIAS[newid]	.3995332	.0504922	7.91	0.000	.3005703	.498496
c.m1#TRAIT[newid]	.9003236	.0512137	17.58	0.000	.7999466	1.000701
av_4_						
m2	2.471133	.1258205	19.64	0.000	2.22453	2.717737

```
          m3 |   2.396825   .1296374    18.49   0.000      2.14274    2.650909
```

	Coef.	Std. Err.	z	P>z	[95% Conf.	Interval]
m4	2.289887	.1223861	18.71	0.000	2.050015	2.52976
m5	1.620394	.1009428	16.05	0.000	1.42255	1.818238
m6	.9282427	.1015746	9.14	0.000	.7291602	1.127325
m7	-.4827052	.124283	-3.88	0.000	-.7262954	-.239115
BIAS[newid]	.2992527	.0502297	5.96	0.000	.2008042	.3977011
c.m1#TRAIT[newid]	1.067942	.0598505	17.84	0.000	.9506373	1.185247
av_5_						
m2	3.669743	.1560156	23.52	0.000	3.363958	3.975528
m3	3.841376	.1687888	22.76	0.000	3.510556	4.172196
m4	2.868381	.1440936	19.91	0.000	2.585963	3.150799
m5	2.054407	.1160215	17.71	0.000	1.827009	2.281805
m6	1.89282	.1162673	16.28	0.000	1.66494	2.1207
m7	.3665844	.1364264	2.69	0.007	.0991935	.6339753
BIAS[newid]	.3857878	.0550991	7.00	0.000	.2777955	.4937802
c.m1#TRAIT[newid]	1.3137	.0776658	16.91	0.000	1.161478	1.465922
av_6_						
m2	2.701899	.1336203	20.22	0.000	2.440008	2.96379
m3	2.689099	.1310565	20.52	0.000	2.432233	2.945965
m4	2.094154	.122243	17.13	0.000	1.854562	2.333746
m5	1.735358	.1007596	17.22	0.000	1.537873	1.932843
m6	1.18929	.0979821	12.14	0.000	.9972481	1.381331
m7	-.7034039	.1343507	-5.24	0.000	-.9667264	-.4400814
BIAS[newid]	.3201085	.0423467	7.56	0.000	.2371105	.4031066
c.m1#TRAIT[newid]	.9419438	.0528914	17.81	0.000	.8382786	1.045609
av_7_						
m2	3.863887	.1782125	21.68	0.000	3.514597	4.213178
m3	4.07216	.1871149	21.76	0.000	3.705422	4.438899
m4	3.267591	.1641134	19.91	0.000	2.945934	3.589247
m5	3.031224	.146897	20.64	0.000	2.743312	3.319137
m6	2.397173	.1421247	16.87	0.000	2.118614	2.675732
m7	.1275834	.1740825	0.73	0.464	-.213612	.4687789
BIAS[newid]	.5289018	.0527634	10.02	0.000	.4254875	.6323161
c.m1#TRAIT[newid]	1.559315	.0951859	16.38	0.000	1.372754	1.745876
av_8_						
m2	3.782128	.1783	21.21	0.000	3.432667	4.13159
m3	2.644992	.163718	16.16	0.000	2.32411	2.965873
m4	2.934417	.161223	18.20	0.000	2.618426	3.250409
m5	2.099819	.1431257	14.67	0.000	1.819298	2.38034
m6	1.506646	.1417629	10.63	0.000	1.228796	1.784496
m7	-.473956	.1603141	-2.96	0.003	-.7881659	-.1597461
BIAS[newid]	.3995131	.0454884	8.78	0.000	.3103574	.4886687
c.m1#TRAIT[newid]	1.835813	.1078859	17.02	0.000	1.624361	2.047266
av_9_						
m2	4.064299	.1691237	24.03	0.000	3.732823	4.395775
m3	3.676914	.1654342	22.23	0.000	3.352669	4.001159
m4	3.232263	.1575273	20.52	0.000	2.923515	3.541011
m5	2.365751	.1331719	17.76	0.000	2.104739	2.626764
m6	1.95898	.1333361	14.69	0.000	1.697646	2.220314
m7	.950899	.1439321	6.61	0.000	.6687972	1.233001

```
BIAS[newid] |    .4461087   .0478944     9.31   0.000     .3522374    .5399801
            |
```

c.m1#TRAIT[newid]	1.59891	.0906614	17.64	0.000	1.421217	1.776603
av_10_						
m2	5.518156	.2608959	21.15	0.000	5.00681	6.029503
m3	5.029499	.2469957	20.36	0.000	4.545396	5.513601
m4	5.03613	.2445514	20.59	0.000	4.556818	5.515442
m5	4.158631	.2189947	18.99	0.000	3.729409	4.587852
m6	4.102615	.2177104	18.84	0.000	3.67591	4.529319
m7	1.854694	.2187674	8.48	0.000	1.425918	2.28347
BIAS[newid]	.5304518	.0569911	9.31	0.000	.4187513	.6421524
c.m1#TRAIT[newid]	1.238602	.1336952	9.26	0.000	.9765646	1.50064
/av_1_						
cut1	1.7283	.0991259			1.534017	1.922583
cut2	2.19442	.1029604			1.992622	2.396219
cut3	3.226344	.1193284			2.992465	3.460224
/av_2_						
cut1	.7800032	.0597545			.6628865	.8971198
cut2	1.112092	.0612209			.9921012	1.232083
cut3	1.995643	.0716595			1.855193	2.136093
/av_3_						
cut1	.5817363	.0708883			.4427977	.7206748
cut2	1.529071	.0785729			1.375071	1.683072
cut3	2.594666	.0949099			2.408646	2.780686
/av_4_						
cut1	.40341	.075572			.2552916	.5515284
cut2	1.327078	.0812645			1.167802	1.486353
cut3	2.573029	.0948771			2.387073	2.758985
/av_5_						
cut1	1.016986	.0962081			.8284218	1.205551
cut2	2.031862	.1040356			1.827956	2.235768
cut3	3.317839	.1207419			3.081189	3.554489
/av_6_						
cut1	1.120652	.0760756			.9715462	1.269757
cut2	1.624013	.0796714			1.46786	1.780166
cut3	2.279752	.0871626			2.108916	2.450588
/av_7_						
cut1	1.584422	.126983			1.33554	1.833304
cut2	2.523425	.1358011			2.25726	2.78959
cut3	3.3227	.1441587			3.040154	3.605245
/av_8_						
cut1	.9284056	.1248161			.6837705	1.173041
cut2	2.459143	.138174			2.188327	2.729959
cut3	3.311602	.1488885			3.019786	3.603418
/av_9_						
cut1	1.160727	.1131206			.9390145	1.382439
cut2	2.109137	.1207691			1.872434	2.34584
cut3	3.463966	.1368648			3.195716	3.732216
/av_10_						
cut1	3.162176	.2157467			2.739321	3.585032
cut2	4.13528	.2203234			3.703454	4.567105
cut3	5.213893	.2312851			4.760582	5.667203
mean(BIAS[newid])	0	(omitted)				

```
  mean(TRAIT[newid])|            0  (omitted)
------------------------------------------------------------------------------
```

```
   var(BIAS[newid])|          1  (constrained)
  var(TRAIT[newid])|          1  (constrained)
------------------------------------------------------------------------------

Group:     2                                         Number of obs = 2,312

Response: av_1_                                      Number of obs = 1,327
Family:   Ordinal
Link:     Probit

Response: av_2_                                      Number of obs = 1,328
Family:   Ordinal
Link:     Probit

Response: av_3_                                      Number of obs = 1,324
Family:   Ordinal
Link:     Probit

Response: av_4_                                      Number of obs = 1,324
Family:   Ordinal
Link:     Probit

Response: av_5_                                      Number of obs = 1,326
Family:   Ordinal
Link:     Probit

Response: av_6_                                      Number of obs = 1,327
Family:   Ordinal
Link:     Probit

Response: av_7_                                      Number of obs = 1,327
Family:   Ordinal
Link:     Probit

Response: av_8_                                      Number of obs = 1,322
Family:   Ordinal
Link:     Probit

Response: av_9_                                      Number of obs = 1,323
Family:   Ordinal
Link:     Probit

Response: av_10_                                     Number of obs = 1,327
Family:   Ordinal
Link:     Probit

------------------------------------------------------------------------------
                   | Coefficient  Std. err.      z    P>|z|     [95% conf. interval]
-------------------+----------------------------------------------------------------
av_1_              |
                m2 |   3.045255    .146489    20.79   0.000     2.758142    3.332368
                m3 |   2.863498   .1410171    20.31   0.000      2.58711    3.139887
                m4 |   2.298565   .1327731    17.31   0.000     2.038334    2.558795
                m5 |   1.967435   .1128217    17.44   0.000     1.746309    2.188562
                m6 |   1.398545    .116863    11.97   0.000     1.169498    1.627593
                m7 |   .5683581   .1491886     3.81   0.000     .2759537    .8607624
                   |
       BIAS[newid] |  -.0166117   .0430374    -0.39   0.700    -.1009635    .0677401
                   |
 c.m1#TRAIT[newid] |   .9309581   .0787886    11.82   0.000     .7765352    1.085381
-------------------+----------------------------------------------------------------
av_2_              |
                m2 |   2.782966   .1219367    22.82   0.000     2.543974    3.021957
                m3 |    2.59733   .1178091    22.05   0.000     2.366428    2.828231
                m4 |    2.12574   .1146923    18.53   0.000     1.900947    2.350533
```

m5 |	1.823478	.0865834	21.06	0.000	1.653777	1.993178
m6 |	1.608219	.09298	17.30	0.000	1.425982	1.790457

m7	.6774317	.1166204	5.81	0.000	.4488598	.9060036
BIAS[newid]	-.0510329	.0390731	-1.31	0.192	-.1276148	.0255489
c.m1#TRAIT[newid]	.5517016	.0527336	10.46	0.000	.4483456	.6550575
av_3_						
m2	2.714612	.1330214	20.41	0.000	2.453895	2.975329
m3	1.644821	.1178975	13.95	0.000	1.413746	1.875896
m4	2.052563	.1186465	17.30	0.000	1.82002	2.285106
m5	1.361059	.0947411	14.37	0.000	1.17537	1.546748
m6	.9955193	.0964128	10.33	0.000	.8065536	1.184485
m7	-.2510729	.1221723	-2.06	0.040	-.4905262	-.0116195
BIAS[newid]	.3995332	.0504922	7.91	0.000	.3005703	.498496
c.m1#TRAIT[newid]	.9003236	.0512137	17.58	0.000	.7999466	1.000701
av_4_						
m2	2.471133	.1258205	19.64	0.000	2.22453	2.717737
m3	2.396825	.1296374	18.49	0.000	2.14274	2.650909
m4	2.289887	.1223861	18.71	0.000	2.050015	2.52976
m5	1.620394	.1009428	16.05	0.000	1.42255	1.818238
m6	.9282427	.1015746	9.14	0.000	.7291602	1.127325
m7	-.4827052	.124283	-3.88	0.000	-.7262954	-.239115
BIAS[newid]	.2992527	.0502297	5.96	0.000	.2008042	.3977011
c.m1#TRAIT[newid]	1.067942	.0598505	17.84	0.000	.9506373	1.185247
av_5_						
m2	3.669743	.1560156	23.52	0.000	3.363958	3.975528
m3	3.841376	.1687888	22.76	0.000	3.510556	4.172196
m4	2.868381	.1440936	19.91	0.000	2.585963	3.150799
m5	2.054407	.1160215	17.71	0.000	1.827009	2.281805
m6	1.89282	.1162673	16.28	0.000	1.66494	2.1207
m7	.3665844	.1364264	2.69	0.007	.0991935	.6339753
BIAS[newid]	.3857878	.0550991	7.00	0.000	.2777955	.4937802
c.m1#TRAIT[newid]	1.3137	.0776658	16.91	0.000	1.161478	1.465922
av_6_						
m2	2.701899	.1336203	20.22	0.000	2.440008	2.96379
m3	2.689099	.1310565	20.52	0.000	2.432233	2.945965
m4	2.094154	.122243	17.13	0.000	1.854562	2.333746
m5	1.735358	.1007596	17.22	0.000	1.537873	1.932843
m6	1.18929	.0979821	12.14	0.000	.9972481	1.381331
m7	-.7034039	.1343507	-5.24	0.000	-.9667264	-.4400814
BIAS[newid]	.3201085	.0423467	7.56	0.000	.2371105	.4031066
c.m1#TRAIT[newid]	.9419438	.0528914	17.81	0.000	.8382786	1.045609
av_7_						
m2	3.863887	.1782125	21.68	0.000	3.514597	4.213178
m3	4.07216	.1871149	21.76	0.000	3.705422	4.438899
m4	3.267591	.1641134	19.91	0.000	2.945934	3.589247
m5	3.031224	.146897	20.64	0.000	2.743312	3.319137
m6	2.397173	.1421247	16.87	0.000	2.118614	2.675732
m7	.1275834	.1740825	0.73	0.464	-.213612	.4687789
BIAS[newid]	.5289018	.0527634	10.02	0.000	.4254875	.6323161
c.m1#TRAIT[newid]	1.559315	.0951859	16.38	0.000	1.372754	1.745876

```
-----------------------------------------+--------------------------------------------------------------------------------------------------------------------------------------------------
av_8_                                    |
```

m2	3.782128	.1783	21.21	0.000	3.432667	4.13159
m3	2.644992	.163718	16.16	0.000	2.32411	2.965873
m4	2.934417	.161223	18.20	0.000	2.618426	3.250409
m5	2.099819	.1431257	14.67	0.000	1.819298	2.38034
m6	1.506646	.1417629	10.63	0.000	1.228796	1.784496
m7	-.473956	.1603141	-2.96	0.003	-.7881659	-.1597461
BIAS[newid]	.3995131	.0454884	8.78	0.000	.3103574	.4886687
c.m1#TRAIT[newid]	1.835813	.1078859	17.02	0.000	1.624361	2.047266
av_9_						
m2	4.064299	.1691237	24.03	0.000	3.732823	4.395775
m3	3.676914	.1654342	22.23	0.000	3.352669	4.001159
m4	3.232263	.1575273	20.52	0.000	2.923515	3.541011
m5	2.365751	.1331719	17.76	0.000	2.104739	2.626764
m6	1.95898	.1333361	14.69	0.000	1.697646	2.220314
m7	.950899	.1439321	6.61	0.000	.6687972	1.233001
BIAS[newid]	.4461087	.0478944	9.31	0.000	.3522374	.5399801
c.m1#TRAIT[newid]	1.59891	.0906614	17.64	0.000	1.421217	1.776603
av_10_						
m2	5.518156	.2608959	21.15	0.000	5.00681	6.029503
m3	5.029499	.2469957	20.36	0.000	4.545396	5.513601
m4	5.03613	.2445514	20.59	0.000	4.556818	5.515442
m5	4.158631	.2189947	18.99	0.000	3.729409	4.587852
m6	4.102615	.2177104	18.84	0.000	3.67591	4.529319
m7	1.854694	.2187674	8.48	0.000	1.425918	2.28347
BIAS[newid]	.5304518	.0569911	9.31	0.000	.4187513	.6421524
c.m1#TRAIT[newid]	1.238602	.1336952	9.26	0.000	.9765646	1.50064
/av_1_						
cut1	1.174051	.0874912			1.002571	1.34553
cut2	2.259924	.1033557			2.05735	2.462497
cut3	3.847255	.1338181			3.584977	4.109534
/av_2_						
cut1	1.010954	.061729			.8899676	1.131941
cut2	2.022537	.0745913			1.876341	2.168733
cut3	2.887824	.0883213			2.714717	3.06093
/av_3_						
cut1	-.9163668	.0769785			-1.067242	-.7654917
cut2	.567571	.0700916			.4301939	.704948
cut3	2.302781	.0881356			2.130038	2.475524
/av_4_						
cut1	-.4083957	.0793879			-.563993	-.2527983
cut2	.6165402	.0781511			.4633669	.7697135
cut3	2.338113	.0947924			2.152323	2.523902
/av_5_						
cut1	.28404	.093281			.1012127	.4668673
cut2	1.35013	.0992851			1.155535	1.544725
cut3	3.06983	.122608			2.829523	3.310138
/av_6_						
cut1	-.6185966	.075179			-.7659447	-.4712484
cut2	.9032123	.0748877			.7564352	1.049989
cut3	2.725718	.1002891			2.529155	2.922281

```
/av_7_               |
             cut1 |   .6063233    .111505                            .3877775    .8248691
```

```
             cut2 |   1.863819   .1262368                             1.6164
                      2.111239
             cut3 |   3.409462   .1472464                           3.120864
                      3.698059
-------------+----------------------------------------------------------------
/av_8_            |
             cut1 |  -.3666432   .1228575                          -.6074396
                     -.1258469
             cut2 |   1.641348    .131622                           1.383373
                      1.899322
             cut3 |   3.266398   .1493769                           2.973624
                      3.559171
-------------+----------------------------------------------------------------
/av_9_            |
             cut1 |   .4510258   .1103646                           .2347152
                      .6673364
             cut2 |   2.352097    .127074                           2.103037
                      2.601158
             cut3 |   3.768693    .145879                           3.482776
                      4.054611
-------------+----------------------------------------------------------------
/av_10_           |
             cut1 |   2.438018   .1700321                           2.104761
                      2.771274
             cut2 |   3.011439   .1899292                           2.639185
                      3.383694
             cut3 |   4.202482   .2126664                           3.785664
                      4.619301
-------------+----------------------------------------------------------------
  mean(BIAS[newid])|          0  (omitted)
 mean(TRAIT[newid])|  -.2469503   .0728365    -3.39   0.001    -.3897071
                     -.1041934
-------------+----------------------------------------------------------------
    var(BIAS[newid])|          1
                          (const
                         rained)
   var(TRAIT[newid])|          1
------------------------------------------------------------------------------
                          (cons
                         trained)

. estat ic

Akaike's information criterion and Bayesian information criterion

-----------------------------------------------------------------------------
       Model |          N   ll(null)  ll(model)      df        AIC
                        BIC
-------------+
-------------
           . |      4,006          .  -19691.35     141   39664.71   40552.38
-----------------------------------------------------------------------------
Note: BIC uses N = number of observations. See [R] BIC note.

.
.
.
.
.
.
end of do-file
```

Web Figure 1

Histograms showing density distribution of vignette ratings for each EPDS item, split by country.

EPDS Item	Cohort	AV-A – Most Severe	AV-B	AV-C	AV-D	AV-E	AV-F – Least Severe
1	BCHADS	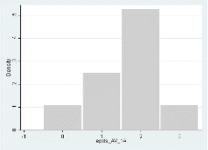	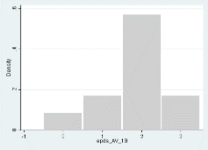	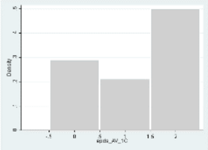	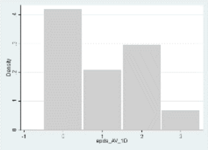	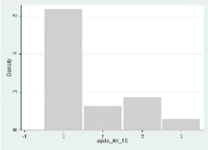	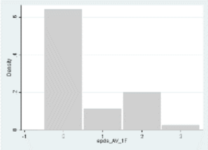
	UKAV	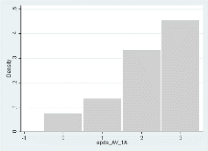	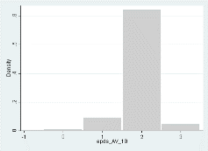	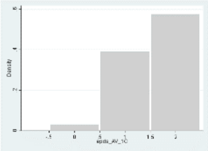	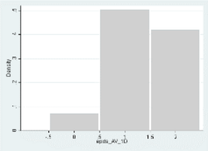	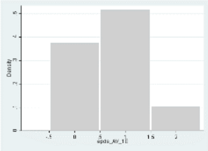	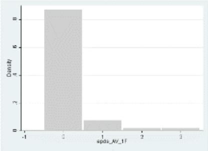
2	BCHADS	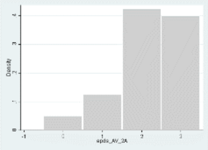	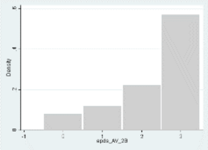	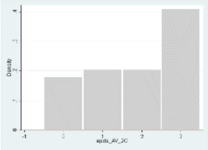	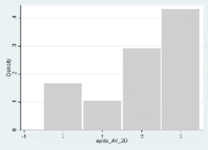	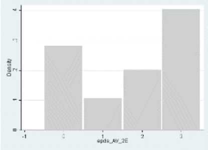	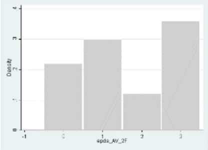
	UKAV	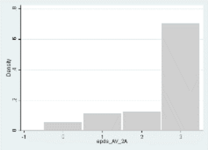	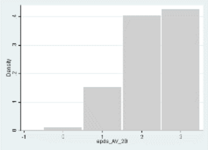	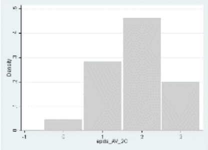	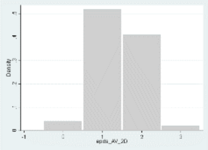	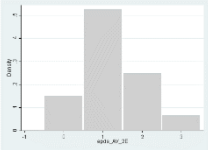	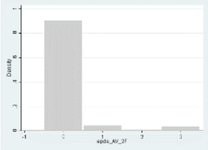
3	BCHADS	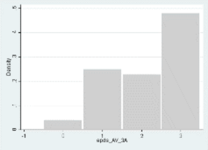	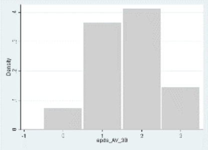	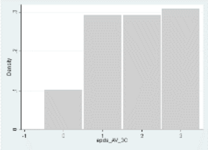	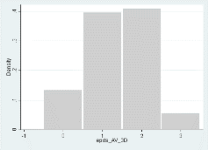	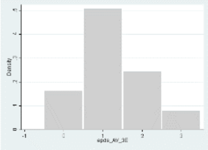	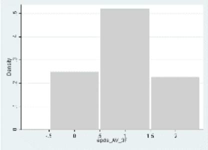

**UKAV**	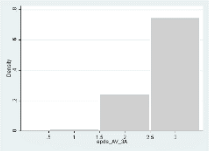	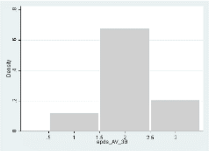	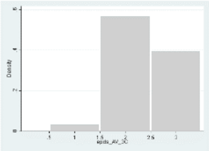	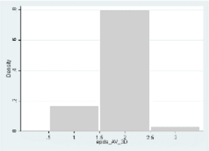	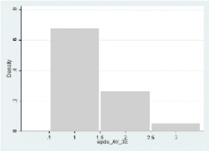	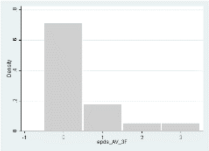

EPDS Item	Cohort	AV-A – Most Severe	AV-B	AV-C	AV-D	AV-E	AV-F – Least Severe
4	BCHADS	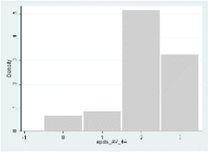	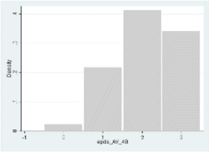	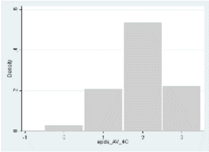	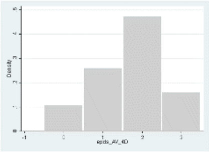	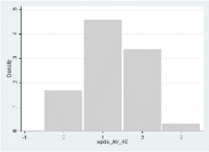	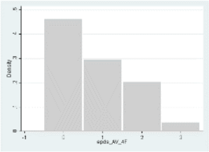
	UKAV	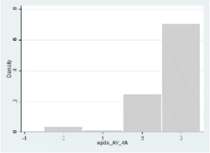	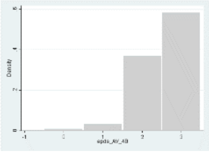	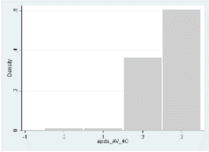	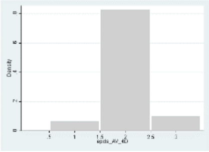	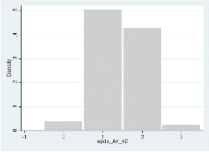	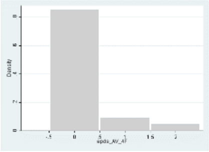
5	BCHADS	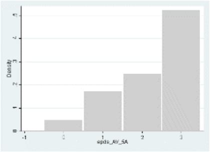	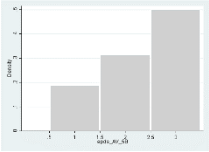	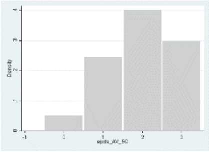	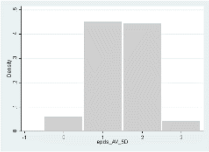	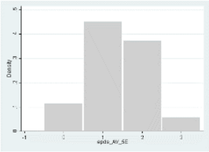	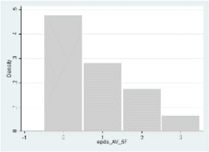
	UKAV	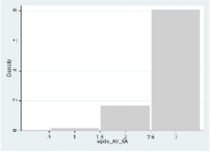	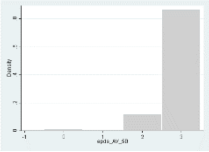	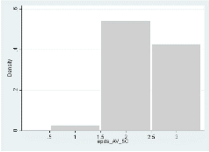	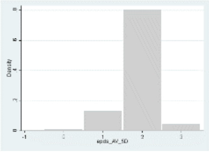	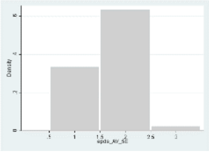	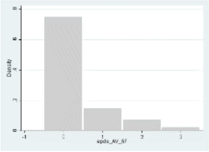

EPDS Item	Cohort	AV-A – Most Severe	AV-B	AV-C	AV-D	AV-E	AV-F – Least Severe
6	**BCHADS**	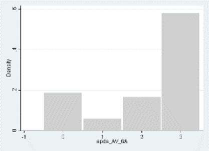	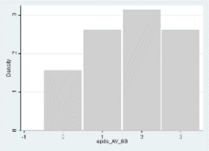	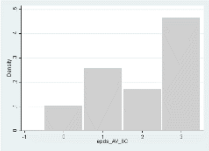	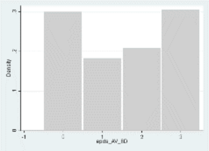	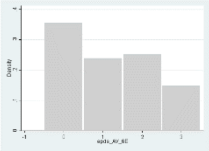	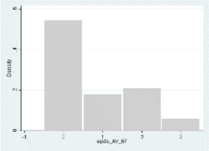
	**UKAV**	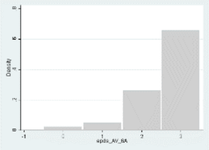	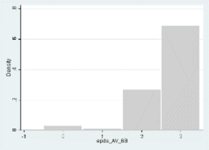	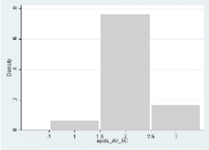	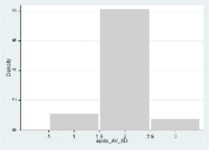	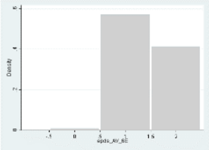	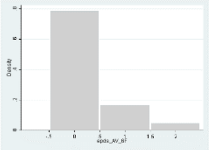
7	**BCHADS**	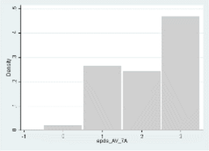	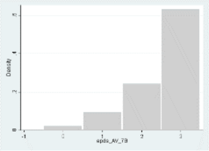	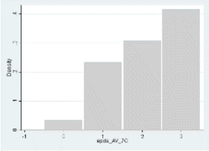	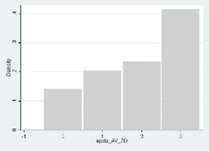	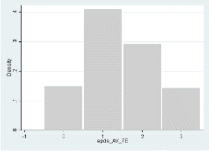	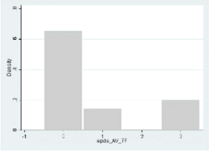
	**UKAV**	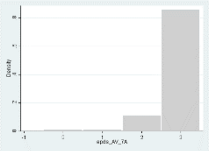	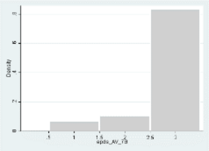	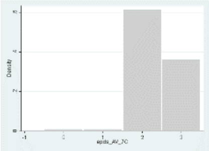	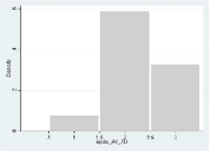	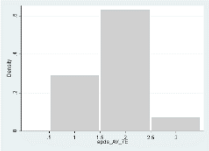	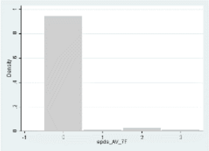
8	**BCHADS**	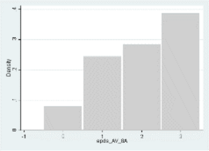	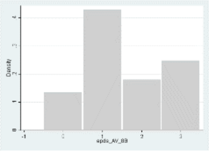	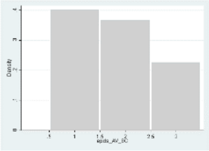	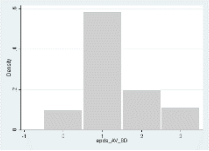	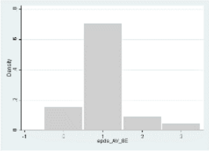	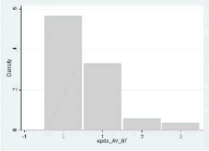
	**UKAV**	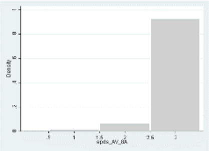	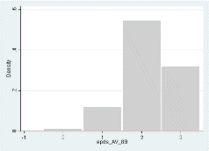	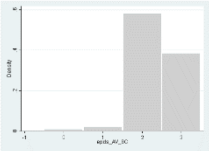	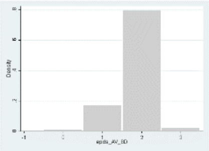	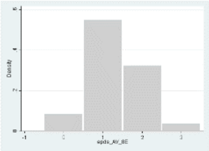	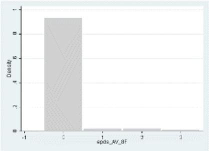

EPDS Item	Cohort	AV-A – Most Severe	AV-B	AV-C	AV-D	AV-E	AV-F – Least Severe
9	BCHADS	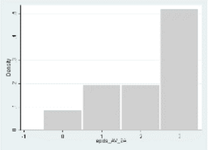	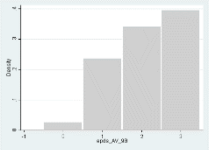	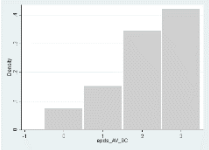	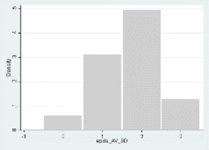	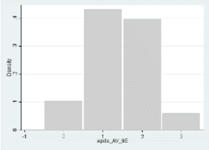	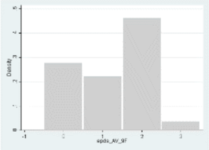
	UKAV	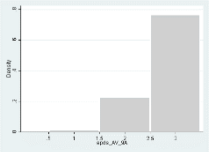	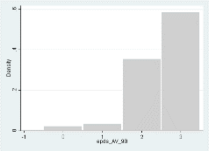	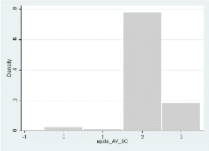	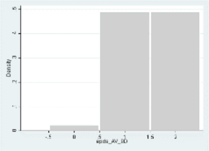	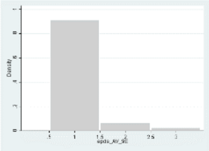	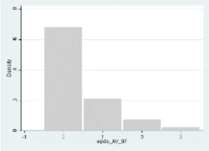
10	BCHADS	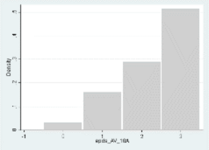	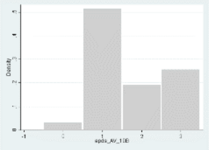	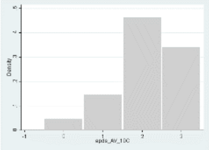	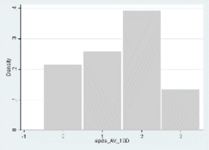	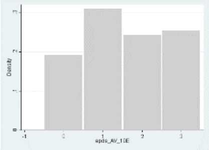	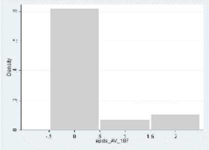
	UKAV	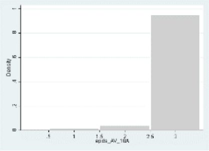	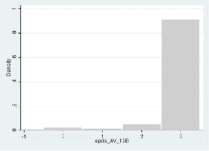	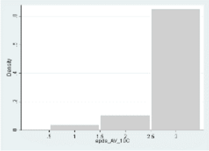	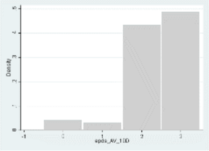	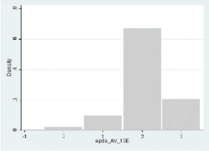	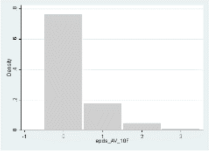

Web Figure 2

Mean AV ratings (0-3) for India (dark grey) and UK (light grey) separately for each EPDS item set of vignettes.

EPDS Item 1

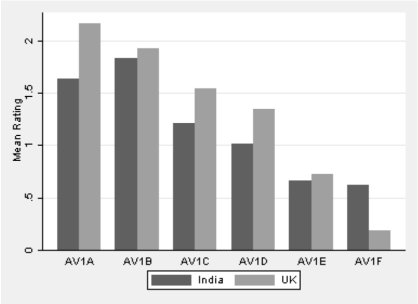

EPDS Item 2

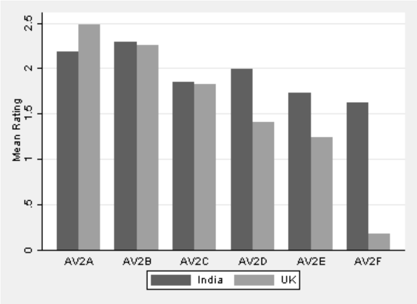

EPDS Item 3

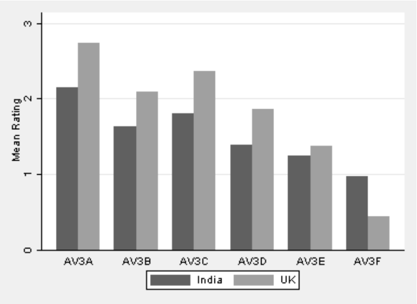

EPDS Item 4

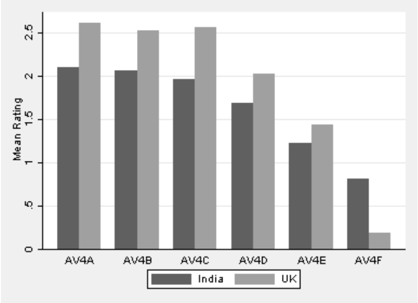

EPDS Item 5

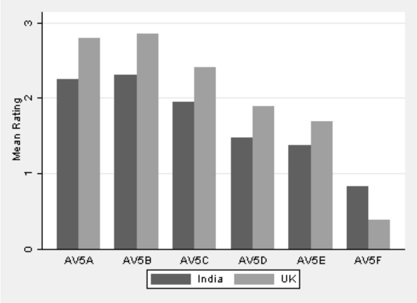

EPDS Item 6

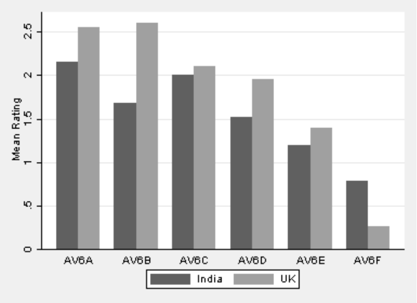

EPDS Item 7

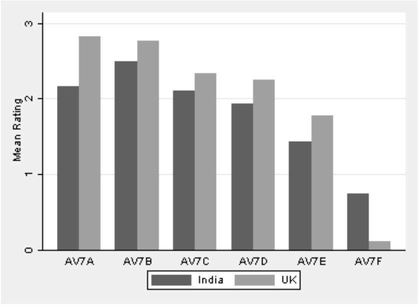

EPDS Item 8

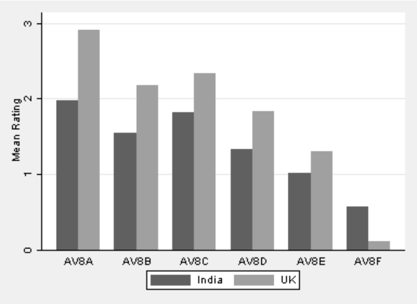

EPDS Item 9

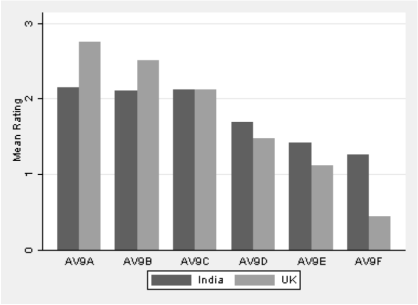

EPDS Item 10

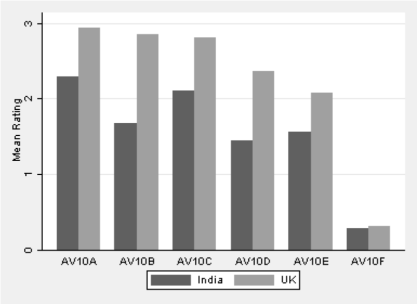

Web Figure S3

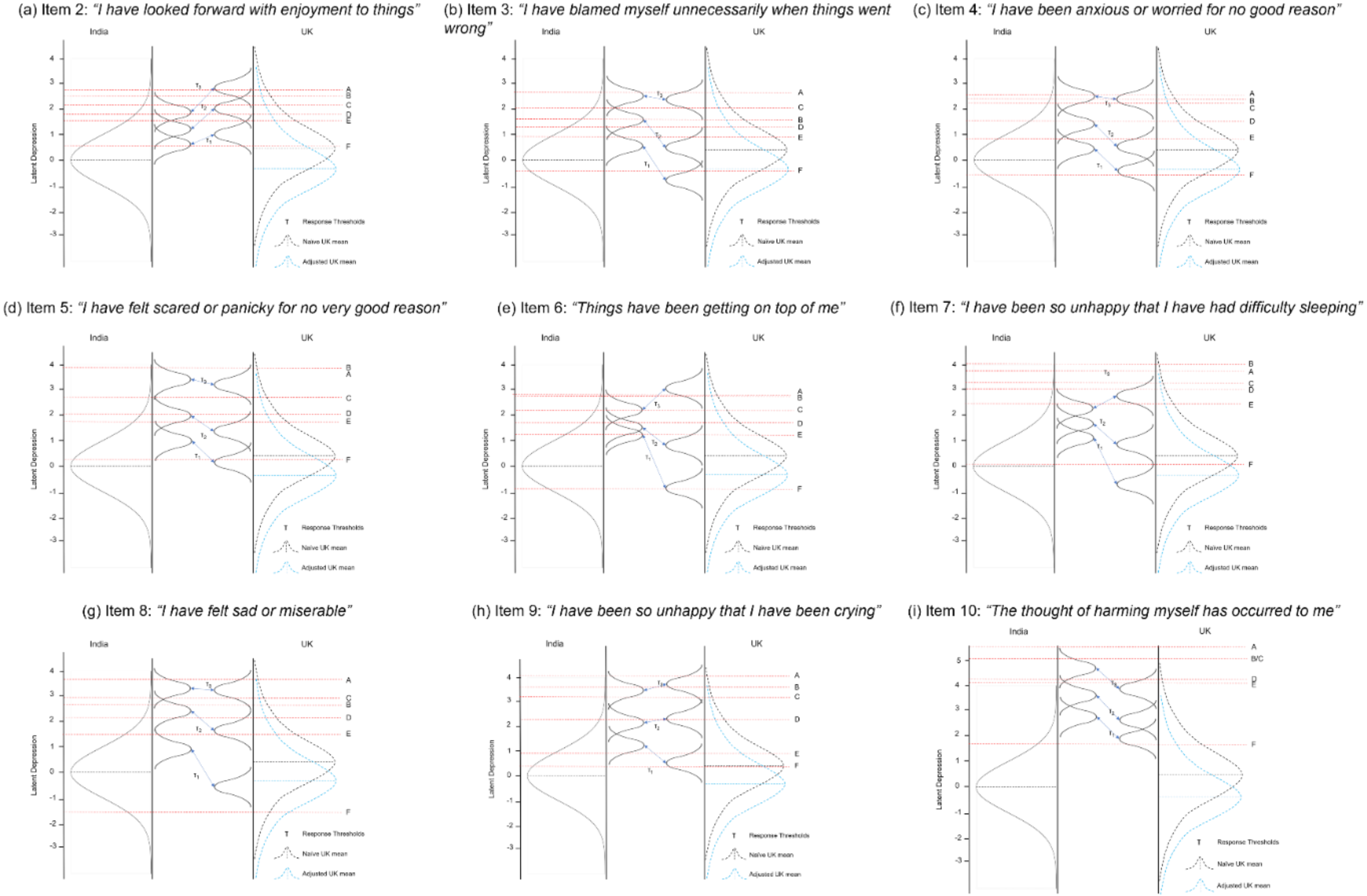

References

1. Au N, Lorgelly PK. Anchoring vignettes for health comparisons: an analysis of response consistency. *Qual Life Res*. 2014;23(6):1721–1731.
2. Cox JL, Holden JM, Sagovsky R. Detection of postnatal depression. Development of the 10-item Edinburgh Postnatal Depression Scale. *Br J Psychiatry*. 1987;150:782–786.
3. Knott RJ, Lorgelly PK, Black N, Hollingsworth B. Differential item functioning in quality of life measurement: an analysis using anchoring vignettes. *Soc Sci Med*. 2017;190:247–255.
4. King G, Murray CJL, Salomon JA, Tandon AJ. Enhancing the validity and cross-cultural comparability of measurement in survey research. *Am Polit Sci Rev*. 2004;98(1):191–207.
